# Supplementary material for: Rhodiola sacra Protects Against Hippocampal Neuronal Apoptosis in Chronic Cerebral Ischemia via SIRT1-Driven Mitochondrial Biogenesis
Source: Curr Neuropharmacol. 2026 Apr 20;24(9):1365–80. doi: 10.2174/011570159X417392251118050138 (PMC13536743; doi:10.2174/011570159X417392251118050138)
Supplement: Supplementary file 1 [file CN-24-9-1365_SD1.pdf]

Send Orders for Reprints to reprints@benthamscience.net

Current Neuropharmacology, 2026, 24, 1365-1380

RESEARCH ARTICLE

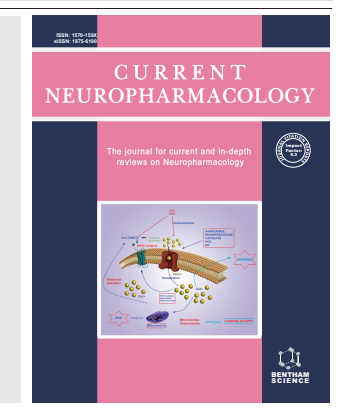

Rhodiola sacra Protects Against Hippocampal Neuronal Apoptosis in Chronic Cerebral Ischemia *via* SIRT1-Driven Mitochondrial Biogenesis

Lingyi Zhou[1,2,#], Jing Jia[3,#], Jinping Li[3], Zhi Yao[2,*], Zheng Fu[3,*] and Shuai Wang[4,*]

[1]Department of Blood Transfusion, Tianjin Medical University General Hospital, Tianjin, 300052, China; [2]Key Laboratory of Immune Microenvironment and Disease (Ministry of Education), State Key Laboratory of Experimental Hematology, The Province and Ministry Co-sponsored Collaborative Innovation Center for Medical Epigenetics, Department of Immunology, Tianjin Institute of Immunology, School of Basic Medical Sciences, Tianjin Medical University, Tianjin, 300070, China; [3]Tianjin Kangzhe Pharmaceutical Technology Development Company, Ltd., Tianjin, 300042, China; [4]Department of Radiation Oncology, Shandong Cancer Hospital and Institute, Shandong First Medical University and Shandong Academy of Medical Sciences, Jinan, Shandong, 250117, China

© 2026 The Author(s). Published by Bentham Science Publisher. This is an open access article published under CC BY 4.0 https://creativecommons.org/licenses/by/4.0/legalcode

ARTICLE HISTORY

Received: June 28, 2025
Revised: September 20, 2025
Accepted: September 24, 2025

DOI:
10.2174/011570159X417392251118050138

Abstract: ***Introduction***: Chronic cerebral ischemia (CCI) induces hippocampal neuronal injury, with mitochondrial dysfunction emerging as a pivotal pathological driver of ischemic brain damage. Enhancing mitochondrial biogenesis (MB) represents a promising reparative strategy to restore neuronal homeostasis. *Rhodiola sacra* (RS), a traditional Tibetan herb, exhibits neuroprotective potential against ischemic injury; however, its underlying mechanisms, particularly its association with MB, remain unclear. This study aims to investigate the protective effects of RS on neuronal apoptosis and mitochondrial dysfunction in CCI rats and oxygen-glucose deprivation (OGD)-exposed cells, with a focus on elucidating the role of SIRT1 in mediating these effects.

Methods: The CCI rat model was established through bilateral common carotid artery occlusion. Spatial learning and memory abilities were evaluated using the Morris water maze. Hippocampal neuronal apoptosis was assessed *via* TUNEL staining, and mitochondrial damage was examined using transmission electron microscopy. Flow cytometry was employed to detect cell apoptosis, mitochondrial membrane potential (MMP), and reactive oxygen species (ROS) levels. Immunofluorescence staining was used to visualize the mitochondrial permeability transition pore (mPTP). Intracellular ATP levels were measured using a luminol-based chemiluminescence assay, and mitochondrial DNA (mtDNA) content was quantified by qPCR. Additionally, small interfering RNA (siRNA) was utilized to verify the critical role of SIRT1 in mediating the protective effects.

Results: RS significantly improved spatial learning and memory, attenuated hippocampal neuronal apoptosis, and increased mitochondrial content in CCI rats. In SH-SY5Y cells exposed to OGD, salidroside, a key active component of RS, reduced apoptosis and restored mitochondrial function, as evidenced by elevated MMP, ATP, and mtDNA levels, along with suppressed ROS and mPTP activity. Mechanistically, salidroside upregulated SIRT1 expression, which correlated with enhanced MB markers. Notably, inhibition of SIRT1 abolished the anti-apoptotic effects and MB activation induced by salidroside.

Discussion: These findings highlight RS as a promising therapeutic agent for ischemic brain injury, targeting SIRT1 to restore MB.

Conclusion: RS mitigates neuronal apoptosis and mitochondrial dysfunction in CCI through SIRT1-dependent MB activation.

Keywords: Chronic cerebral ischemia, *Rhodiola sacra*, SIRT1, mitochondrial biogenesis, apoptosis, SIRT1 expression.

*Address correspondence to these authors at the Key Laboratory of Immune Microenvironment and Disease (Ministry of Education), State Key Laboratory of Experimental Hematology, The Province and Ministry Co-sponsored Collaborative Innovation Center for Medical Epigenetics, Department of Immunology, Tianjin Institute of Immunology, School of Basic Medical Sciences, Tianjin Medical University, Tianjin, 300070, China; E-mail: yaozhi@tmu.edu.cn (Z.Y.); Tianjin Kangzhe Pharmaceutical Technology Development Company, Ltd., Tianjin, 300042, China; E-mail: fuzhengcn@vip.sina.com (Z.F.); Department of Radiation Oncology, Shandong Cancer Hospital and Institute, Shandong First Medical University and Shandong Academy of Medical Sciences, Jinan, Shandong, 250117, China; E-mail: wsh5635@163.com (S.W.)
[#]*These authors have contributed equally to this work.*

Current Neuropharmacology

1. INTRODUCTION

Chronic cerebral ischemia, a condition characterized by reduced or interrupted blood flow to various brain regions, results in insufficient oxygen supply to neurons. It is prevalent among the elderly and is a leading cause of high disability and mortality worldwide [1]. Recent studies have demonstrated that chronic cerebral ischemia induces persistent cognitive impairment and neuronal damage, particularly in the hippocampus [2]. This damage is closely associated with the disruption of mitochondrial homeostasis in the brain, and the restoration of MB has been shown to mitigate ischemic cerebral damage [3, 4]. Current primary treatments, including surgery and thrombolytic drugs, primarily focus on removing thrombi that block blood vessels but do not address the neuronal damage caused by ischemia [5-7]. Although neuroprotective agents have demonstrated efficacy in animal models of ischemic brain injury, their clinical translation has been hampered by unpredictable toxicity, suboptimal blood-brain barrier penetration, and interspecies pharmacological disparities [8]. Therefore, repurposing clinically approved drugs with established safety profiles and known pharmacokinetic properties represents a pragmatic strategy to circumvent these barriers.

Recently, several traditional natural medicines have been reported to exhibit protective effects in the prevention and treatment of various diseases, with higher acceptability and fewer side effects. *Rhodiola sacra*, a traditional natural plant pharmaceutical, is primarily cultivated in the high-altitude Tibetan plateau and is known for its strong environmental adaptability and vitality [9, 10]. Traditionally, *Rhodiola sacra* has been used to prevent and treat high-altitude sickness, dizziness, myocardial infarction, and cerebrovascular diseases [10-13]. A recent study demonstrated that *Rhodiola sacra*, in combination with exercise, enhances mitochondrial quality control, including the activation of mitophagy, mitochondrial dynamics, and biogenesis, in mice subjected to exhaustive exercise[14]. Salidroside, a phenylpropanoid glycoside, is one of the most important bioactive components isolated from *Rhodiola sacra*. Recent studies have shown that salidroside has potential neuropharmacological effects, including improving cognitive function, alleviating Parkinson's disease, Alzheimer's disease, stroke, and brain hypoxia, primarily through the protection of neuronal mitochondrial function [15-20]. However, the underlying mechanisms of its effects in treating chronic cerebral ischemia remain unclear.

Sirtuins are a family of protein deacetylases that regulate numerous cellular pathways, including cell proliferation, differentiation, apoptosis, and metabolism [21]. Among these, Silent information regulator 1 (SIRT1) is a nicotinamide adenine dinucleotide-dependent histone deacetylase that plays a crucial role in MB and mitochondrial function [22-24]. Peroxisome proliferator-activated receptor-γ coactivator- 1α (PGC-1α), a transcriptional coactivator integral to energy metabolism, is a major regulator of mitochondrial biogenesis [25, 26]. SIRT1 regulates the transcription of PGC-1α and other mitochondrial-related genes through promoter deacetylation [23, 24, 27]. Emerging evidence suggests that activation of the AMPK/SIRT1/PGC-1α pathway may mitigate aging-related cognitive decline [28]. Upregulation of SIRT1/PGC-1α signaling has been shown to improve cardiomyocyte apoptosis induced by ischemia/reperfusion [29]. Conversely, inhibition of PGC-1α and SIRT1 exacerbates cell death in neonatal hypoxic-ischemic brain injury [30]. While these studies have established the direct role of SIRT1 in brain function, the mechanisms underlying SIRT1's involvement in chronic cerebral ischemia remain unclear.

The objective of this study was to investigate whether RS attenuates CCI-induced hippocampal neuronal apoptosis through SIRT1-driven MB, addressing critical gaps in current research. Specifically, while existing ischemic therapies primarily target vascular recanalization but fail to repair neuronal damage, the precise mechanisms by which RS regulates mitochondrial homeostasis in CCI remain uncharacterized, and the role of SIRT1 in CCI-induced neuronal apoptosis is undefined, despite its established function in MB. Thus, we aimed to validate the neuroprotective effects of RS and its active component, salidroside, on mitochondrial dysfunction and apoptosis in both CCI rats and OGD-injured neurons, to determine whether SIRT1 mediates RS-induced MB activation *via* the PGC-1α pathway, and to provide preclinical evidence supporting the repurposing of RS as a neuron-targeted therapy for ischemic brain injury.

2. MATERIALS AND METHODS

2.1. Drugs

A highly pure extract from the root of *Rhodiola sacra* (RS) was purchased from Xizang Nordicon Pharmaceutical Corporation (Lhasa, Xizang, China). HPLC-MS analysis revealed that salidroside ($C_{14}H_{20}O_7$), as the main active ingredient of RS, accounted for 2.5%. The preparation and inspection of the RS extract complied with the 2015 Chinese Pharmacopoeia (inspection report number C1051612067). The extract powder was dissolved in distilled water at a concentration of 180 mg/ml. For the cell culture, salidroside (molecular formula: $C_{14}H_{2}0O_7$; molecular weight: 300.31; PubChem CID: 159278) (98% purity) was obtained from Sigma Aldrich (St. Louis, Missouri, USA) and dissolved in the blank DMEM at an original concentration of 50 mg/ml.

2.2. Experimental Design

Sprague-Dawley rats, aged 6-8 weeks and weighing 240 ± 20 g, were purchased from Beijing Vital River Laboratory Animal Technology Co., Ltd. (Beijing, China). All rats were randomly assigned to three groups: sham group (sham-operated rats treated with normal saline); CCI group (chronic cerebral ischemia rats treated with normal saline); and RS group (chronic cerebral ischemia rats treated with *Rhodiola sacra*). The rats were housed under standard laboratory conditions, with a temperature of 23 ± 2°C, a humidity of 50 ± 10%, and a 12-hour light-dark cycle, and had free access to standard feed and tap water.

The experiment was conducted after 1 week of adaptive feeding. The rats were administered *Rhodiola sacra* or normal saline at a dose of 1.8 g/kg per day (equivalent to 45 mg/kg of salidroside). The gavage was initiated one day before the establishment of the CCI rats model and continued until the termination of the experiment, as described in a

previous study [3]. The *Rhodiola sacra* dose was determined based on our preliminary experiments. During the last 6 days of the experiment, spatial learning and memory capabilities were assessed 1 hour after administration of *Rhodiola sacra*. All animal procedures were performed in accordance with the Public Health Service Guide Approved Procedures for the Care and Use of Laboratory Animals and approved by the Animal Ethics Committee of Tianjin Medical University, Tianjin, China.

2.3. Chronic Cerebral Ischemia Rat Model

The chronic cerebral ischemia (CCI) model of rats was established by bilateral carotid artery occlusion [31-33]. Briefly, rats were anesthetized with 10% chloral hydrate (350 mg/kg IP). After incision along the midline of the abdomen, the bilateral common carotid arteries were exposed. We then gently separated the carotid sheath and the vagus nerve. In the CCI and RS groups, the two arteries were ligated with a 5-0 silk suture. Rats in the sham-operated group received the same operation but the carotid arteries were not ligated. During the operation, the rats' body temperature was maintained at 37°C using a heat pad until they recovered to thermal homeostasis, after which they were returned to their home cages.

2.4. Morris Water Maze

Between the 28th and 34th day after the CCI model establishment, the rats were subjected to a Morris water maze (MWM) experiment to assess their spatial learning and memory capabilities [34-37]. The MWM system consists of a circular tank with a diameter of 120 cm and a height of 50 cm, containing water with a temperature of 22 ± 1°C and a depth of 25 cm. To prevent rats from seeing the underwater platform, titanium dioxide was added to the water to make it opaque.

MWM test includes place navigation and the spatial probe test. Place navigation: The rats were trained for five consecutive days. The rats were sequentially led down into the water from four different quadrants of water entry points. The time required for the rats to find the platform from entering the water was recorded, that is, the escape latency. The rats are considered to have found the platform by staying on it for 2 seconds. If the rat fails to find the platform after 90 seconds, it will be guided to the platform and stayed for 10 seconds to become familiar with the environment; the escape latency is considered to be 90 seconds. After the experiment, the rats were towel-dried and put back in their cages to rest. Investigators who conducted the MWM test were blind to the treatment groups. Spatial probe test: The platform was then removed 24 hours after the last training session. The rats were placed into the water at the opposite point to the target quadrant and allowed to swim freely for 90 seconds in the tank. The swimming tracks of mice were recorded, and the percentage of swimming distance and time in the target quadrant, as well as the number of platform area crossings, were calculated as an assessment of spatial memory.

2.5. HE Stain

After the rats had completed the behavioral tests, their brains were transcardially perfused with 4% paraformaldehyde. Paraffin sections (4 um thick) of brain tissue slices at the level of the hippocampus were cut in the rats and stained with hematoxylin and eosin dye. Viable neuronal cells that did not show ischemic damage (pyknosis or shrinkage) in the hippocampal CA1 areas were counted at three different areas for 433 um lengths each and were expressed as averaged values (n/mm). We implemented strict, blinded-evaluation protocols to eliminate potential bias. In HE-stained sections, only neurons with intact nuclei and clearly visible nucleoli were counted as viable, while pyknotic or shrunken cells were excluded.

2.6. TUNEL Stain

Brain sections of the hippocampus were evaluated for the detection of cleavage of genomic DNA with terminal deoxynucleotidyl transferase-mediated dUTP-biotin *in situ* nick end labeling (TUNEL) kit (*In Situ* Cell Death Detection Kit POD, Roche, Basel, Switzerland). The sections were counterstained with hematoxylin. Positively stained cells were counted in three different hippocampal CA1 areas and expressed as averaged values (n/mm). Apoptotic cells were observed under the microscope, and the apoptotic index (number of apoptotic cells/total number of cells × 100%) was calculated. Apoptotic cells are characterized by chromatin condensation, marginalization, nuclear membrane lysis, and chromatin segmentation into blocks or inferred bodies. We implemented strict, blinded-evaluation protocols to eliminate potential bias. In TUNEL staining, apoptotic cells were identified based on characteristic morphological features (chromatin condensation) combined with positive brown staining, with background signal normalized using adjacent non-immune sections as negative controls. To ensure quantification accuracy, we employed ImageJ software with standardized threshold settings for all image analyses, and only cells meeting both morphological and staining intensity criteria were counted as positive.

2.7. Transmission Electron Microscopy (TEM)

The hippocampus of the rats was quickly removed and cut into 1 mm x 1 mm x 1 mm tissue samples. Tissue samples were fixed in 2.5% glutaraldehyde buffer (Sigma, USA) at 4°C, then incubated with osmium tetroxide (OsO_4) and sliced into 1 μm sections. Tissue sections were stained with uranyl acetate and double-stained with lead citrate. The ultrastructure of mitochondrial morphology was observed and imaged using a transmission electron microscope (Olympus, Tianjin, China). We implemented strict, blinded-evaluation protocols to eliminate potential bias. For quantification of mitochondrial content in TEM images, we employed a grid-based stereological approach across three non-overlapping fields per sample, with mitochondrial profiles that met intact cristae and membrane integrity criteria included in the counts. All quantitative data were subjected to inter-rater reliability testing (Cohen's kappa > 0.85), and discrepancies were resolved through consensus review with a third blinded investigator.

2.8. Protein Extraction and Western Blot Analysis

After completing the behavior tests, the rats were transcardially perfused with 4% sterile PBS. The hippocampus

was rapidly isolated and frozen in liquid nitrogen. For tissue protein extraction, the hippocampus tissues were lysed with cold RIPA (radioimmunoprecipitation assay) lysis buffer (Millipore, Billerica, MA, USA) containing complete protease inhibitors (Roche, Basel, Switzerland) at a ratio of 150 μL/5 mg, ground on ice, and incubated on ice for 60 minutes with rotation. For protein extraction from cells, whole-cell lysates were prepared using cold RIPA lysis buffer (Millipore, Billerica, MA, USA) supplemented with complete protease inhibitors (Roche, Basel, Switzerland). The lysates were sonicated on ice, followed by centrifugation for 20-30 minutes (4°C) at 12000g. The supernatant was collected and boiled. The protein concentrations of hippocampus extracts and cell extracts were determined by the BCA Protein Assay Kit (Thermo Fisher Scientific, Waltham, MA, USA). Western blotting was performed as previously described. Briefly, The following antibodies to PGC-1α (Abcam), NRF1 (Abcam), TFAM (Abcam), Caspase 3 (Cell Signaling), Bcl-2 (Cell Signaling), Bax (Cell Signaling), SIRT1 (Cell Signaling) and β -actin (Sigma Aldrich) were applied for protein detection by combination with an HRP-conjugated anti-rabbit IgG or anti-mouse IgG secondary antibody (Sigma Aldrich), which were detected by Immobilon Western Chemiluminescent HRP Substrate (Millipore, Billerica, MA, USA) and exposure to Tanon 6200 Luminescent Imaging Workstation (Tanon Science & Technology Co., Ltd., Shanghai, China).

2.9. High Performance Liquid Chromatography Analysis of *Rhodiola sacra*

For the preparation of the reference solution, 7.01 mg of salidroside (Sigma Aldrich, St. Louis, Missouri, USA) was accurately weighed and dissolved in 50% methanol to make 25 mL. For *Rhodiola sacra* preparation, 0.5 g of *Rhodiola sacra* powder was dissolved in 50 mL of methanol, sonicated (power: 160 watts, frequency: 40 kHz) for 30 minutes, mixed, and filtered. The reference solution (10 μL) and *Rhodiola sacra* solution (10 μL) were injected into the liquid chromatograph and measured at a wavelength of 275 nm by Standard Testing Lab (Shanghai, China). The chromatographic conditions are methanol-water (15:85) as the mobile phase and 275nm as the detection wavelength.

2.10. Oxygen-glucose Deprivation Cell Model

The human neuroblastoma cell line SH-SY5Y (BeNa Culture Collection, BNCC, Beijing, China) were routinely cultured in Dulbecco's modified Eagle's medium (DMEM) (Gibco, USA) with 10% (v/v) fetal bovine serum (FBS), 10 U/ml penicillin and 10 U/ml streptomycin at 37°C in a humidified atmosphere containing 5% CO_2 and 95% air. Before oxygen glucose deprivation (OGD) treatment, SH-SY5Y cells grown to 60-70% confluence were pre-treated with various concentrations of salidroside (Sigma Aldrich, St. Louis, Missouri, USA) dissolved in glucose-free DMEM for 24 hours. For OGD treatment, cells were washed with glucose-free DMEM (Gibco, USA) and refreshed with deoxygenated glucose-free DMEM. Cells were then immediately transferred to an anaerobic bag (Biomerieux, France) containing a gas mixture of 21% CO_2, 78% N2, and less than 0.1% O2, and incubated at 37°C for 4 hours [38-40]. After oxygen-glucose deprivation, cells were re-cultured in glucose-containing DMEM under normoxic conditions in a 5% CO_2 incubator for various time periods. The control groups were given the same procedure but were cultured in glucose-containing DMEM under normoxic conditions.

2.11. RNA Interference

Small-interfering RNA (siRNA) targeting SIRT1 in mice and a scrambled control siRNA were synthesized by GenePharma (Shanghai, China). SH-SY5Y cells were seeded in 6-well plates the day before. When grown to 60-70% confluence, the cells were refreshed with blank DMEM and transfected with 25pmol SIRT1 siRNA or scramble siRNA using Lipofectamine RNAiMAX reagent (Invitrogen, Carlsbad, California, USA). After a six-hour transfection, the medium was replaced with normal DMEM. The efficiency of SIRT1 knockdown was then assessed 48 hours after transfection by Western blotting using antibodies against SIRT1 (Cell Signaling). The experiments were performed 48 hours after transfection.

2.12. MTS Assay

For MTS (3-(4,5-dimethylthiazol-2-yl)-5-(3-carboxymethoxyphenyl)-2-(4-sulfopheny l)-2H-tetrazolium) assay [41, 42], SH-SY5Y cells were seeded in 96-well plates at a density of 6000 cells/well. Within 2 hours after OGD treatment, 20 ul of 0.5 mg/ml MTS reagent (Promega, Madison, Wisconsin, USA) was added to each well. The cells were incubated at 37°C for 2 hours, and then the absorbance was measured at a wavelength of 490nm using a μQuant Universal Microplate Spectrophotometer (Bio-Tek Instruments, Winooski, USA). The percent viability was compared with the control group. Experiments were performed in triplicate on three independent assays.

2.13. Lactate Dehydrogenase (LDH) Assay

For the LDH assay, SH-SY5Y cells were seeded in 96-well plates at a density of 6000 cells/well [43-45]. Within 2 hours after OGD treatment, 50 μL of cell culture supernatants was transferred to a fresh 96-well plate for detection of lactate dehydrogenase activity using a CytoTox-96 Non-Radioactive Cytotoxicity Assay Kit (Promega, Madison, Wisconsin, USA). Cells pretreated with 10X lysis solution for 45 minutes were used as the maximum LDH release control. Then, the reactive solution was added to each well and incubated for 30 minutes at room temperature. The absorbance was recorded using a μQuant Universal Microplate Spectrophotometer (Bio-Tek Instruments, Winooski, USA) at a 490-nm wavelength within 1 hour after adding the stop solution. The percent cytotoxicity was calculated as the experimental LDH Release (OD_{490}) divided by the maximum LDH Release (OD_{490}). Experiments were performed in triplicate on three independent tests.

2.14. Apoptosis Assay

Apoptosis rate was measured by flow cytometry using a PE Annexin V Apoptosis Detection Kit (BD, Franklin Lakes, New Jersey, USA). Within 24 hours after OGD treatment, SH-SY5Y cells were washed twice with cold PBS and resus-

pended in Binding Buffer at a concentration of 1×10^6 cells/ml. Next, PE Annexin V (5 μL) and 7-AAD (5 μL) were added to 100 μL of cells (1×10^5 cells) in a 5 mL culture tube. After 15 minutes of incubation in the dark, the cells were analyzed by flow cytometry within 1 hour.

2.15. RNA Extraction and qPT-PCR

Total RNA was isolated using Trizol reagent (Invitrogen, Carlsbad, California, USA) within 48 hours after OGD exposure. Total RNA (2 μg) was reverse transcribed into the first-strand cDNA using the RevertAid First Strand cDNA Synthesis Kit (Thermo Fisher Scientific, Waltham, MA, USA). Quantitative real-time PCR was performed with the PowerUP SYBR green Master Mix (Applied Biosystems, Waltham, MA, USA) on a 7900 Fast Real-Time PCR System (Roche, Basel, Switzerland). The PCR cycling conditions were 95°C for 5 min, followed by 45 cycles of 95°C for 20 s, 60°C for 20 s, 72°C for 20 s, and 78°C for 1 s. GAPDH was used as the endogenous control. The data were shown as $2^{-\Delta\Delta Ct}$ values, which represented the average of at least three independent experiments. Sequences of the RT-PCR primers (Forward and Reverse) are shown in Table **S1**.

2.16. Measurements of Mitochondrial DNA

For analysis of mitochondrial DNA levels (mtDNA), total DNA was extracted by the Total DNA Extraction Kit (TIANGEN, Beijing, China) within 48 hours after OGD exposure. Relative levels of mtDNA were determined by quantitative real-time PCR (qPCR) assay. Genes encoding 16S rRNA and HK2 were selected to evaluate the relative copy number of mitochondrial DNA (mtDNA) and nuclear DNA (nDNA), which are located in the stable fraction of mtDNA and nDNA. The reactions were performed using the PowerUP SYBR Green Master Mix (Applied Biosystems, Waltham, MA, USA) on a LightCycler 96 Real-Time PCR System (Roche, Basel, Switzerland). Relative mtDNAcn was calculated by the ratio of the 16S rRNA to HK2. The data were shown as $2^{-\Delta\Delta Ct}$ values, which represented the average of at least three independent experiments.

2.17. Reactive Oxygen Species (ROS) Assay

The levels of intracellular ROS were measured using a flow cytometer with the Total ROS Assay Kit (Invitrogen, Carlsbad, California, USA) at 520 nm [3, 46]. Briefly, within 2 hours after OGD treatment, SH-SY5Y cells were harvested by trypsinization and re-suspended in PBS at a density of 1×10^6 cells/ml. The ROS assay stain solution, diluted with DMSO, was used to label the cells. After incubation for 60 minutes at 37°C in a 5% CO_2 incubator, the median fluorescence intensity (MFI) was evaluated using flow cytometry. Experiments were performed in triplicate on three independent assays.

2.18. Assay of Malondialdehyde (MDA) and Superoxide Dismutase (SOD) Levels

The levels of intracellular MDA and SOD were determined with the Lipid Peroxidation Assay Kit (Sigma Aldrich, St. Louis, Missouri, USA) and SOD Determination Kit (Sigma Aldrich, St. Louis, Missouri, USA) respectively [47, 48]. Briefly, within 2 hours after exposure to OGD, SH-SY5Y cells were harvested by trypsinization and sonicated. The cell lysates were centrifuged to collect the supernatant. According to the manufacturers' instructions, the absorbance was measured at 532 nm and 450 nm to analyze the contents of MDA and SOD in the respective supernatants. Experiments were performed in triplicate on three independent assays.

2.19. Determination of ATP Levels

The intracellular ATP levels were determined by Glomax 96 Microplate Luminometer (Promega, Madison, Wisconsin, USA) using CellTiter-Glo® 2.0 Assay (Promega, Madison, Wisconsin, USA) [49, 50]. SH-SY5Y cells were seeded in 96-well plates with opaque plates at a density of 6000 cells/well. Within 2 hours after OGD treatment, the CellTiter-Glo 2.0 Reagent, equal to the volume of cell culture medium, was added to each well. Then, the contents were mixed using an orbital shaker for 2 minutes to induce cell lysis and incubated at room temperature for 10 minutes to stabilize the luminescent signal. The luminescence of cells was recorded. Experiments were performed in triplicate on three independent assays.

2.20. Measurements of Mitochondrial Membrane Potential (MMP)

The changes in MMP were assayed using a flow cytometer with rhodamine 123 (Invitrogen, Carlsbad, California, USA) [51, 52]. Briefly, within 2 hours after OGD treatment, SH-SY5Y cells were washed three times with cold PBS, and rhodamine 123 (10ug/ml) was added to the cells (1×10^6 cells/ml). After 15 minutes of incubation in the dark at 37°C, the median fluorescence intensity (MFI) was detected by flow cytometry. Experiments were performed in triplicate on three independent assays.

2.21. Assay of Mitochondrial Permeability Transition Pore (mPTP)

The state of mitochondrial permeability transition pore, which serves as a key regulator of mitochondrial homeostasis, was measured using the mPTP with red fluorescence (TMRM) Detection Kit (HaLin Biology, Shanghai, China) [53, 54]. SH-SY5Y cells were seeded in the 24-well plates at a density of 2000 cells/well. Within 2 hours after OGD treatment, cells were washed three times with the cleaning solution. The dyeing mixture was added to each well, followed by incubation for 20 minutes at 37°C in the incubator. Then, the cells were rinsed once in a slightly diluted cleaning solution and immediately observed under an inverted fluorescence microscope (Carl Zeiss AG, Jena, Germany) with an excitation wavelength of 540 nm and an emission wavelength of 580 nm. The mean fluorescence intensity was analyzed by Image J software (Rawak Software Inc., Stuttgart, Germany).

2.22. Statistical Analysis

Data are presented as the mean ± SD. Each experiment was performed in triplicate, and at least three independent experiments were analyzed. Analysis of variance (ANOVA)

was used to compare the differences between groups, and data from the MWM experiment were analyzed using Two-way repeated measures ANOVA for statistical significance with SPSS 18.0 statistical software (SPSS, Chicago, Illinois, USA). Values of $p<0.05$ were regarded as statistically significant.

3. RESULTS

3.1. *Rhodiola sacra* Improves Spatial Learning and Memory in CCI Rats

We first employed high-performance liquid chromatography (HPLC) analysis at 275 nm wavelength to identify salidroside (the major active constituent) in *Rhodiola sacra*. The sample exhibited a distinct chromatographic peak at a retention time of 9.401 min, matching the salidroside standard, with no interference observed from the negative control. Under established chromatographic conditions, the salidroside content in *Rhodiola sacra* powder was determined to be 2.5% (Fig. **S1**).

To investigate the neuroprotective effects of *Rhodiola sacra* on hippocampus-dependent spatial cognition, we conducted Morris water maze testing [55]. Following CCI injury in rats (Fig. **1A**). CCI rats exhibited significant impairments in spatial learning and memory, as evidenced by longer escape latencies to locate the hidden platform during training trials, reduced time and distance spent in the target quadrant, and fewer platform area crossings during the probe test compared to sham rats (Fig. **1B-F**). Notably, *Rhodiola sacra* treatment significantly enhanced both spatial learning and memory in CCI rats. Specifically, *Rhodiola sacra* not only reduced escape latencies during training trials but also increased the time and distance spent in the target quadrant and the number of platform area crossings during the probe test (Fig. **1B-F**). Although *Rhodiola sacra*-treated rats (RS group) required more time to locate the hidden platform during training compared to sham rats, the neuroprotective effects of *Rhodiola sacra* were so pronounced that no significant differences were observed between the RS group and sham group in terms of time and distance in the target quadrant or platform area crossings during the probe test (Fig. **1B-F**). Collectively, these results demonstrate that *Rhodiola sacra* effectively protects the rat hippocampus from CCI-induced spatial learning and memory impairments.

3.2. *Rhodiola sacra* Mitigates Hippocampal Neuronal Injury in CCI Rats

To evaluate the *in vivo* neuroprotective effects of *Rhodiola sacra* on CCI, rats were administered *Rhodiola sacra* intragastrically before the establishment of the CCI model and continued throughout the experimental period. Following the water maze test, brain tissues were collected for pathological examination. In the CCI rats, significant degeneration, apoptosis, and loss of neuronal cells were observed in the hippocampal CA1 region. The number of viable neuronal cells (those not showing ischemic damage) in the CCI group (109.78 ± 20.68/mm) was significantly reduced compared to the sham group (175.83 ± 9.44/mm). However, in rats treated with *Rhodiola sacra*, hippocampal neuronal damage was attenuated, and the number of intact neurons in the CA1 region was significantly higher (159.82 ± 18.18/mm) than in the CCI group (Figs. **2A** and **B**).

The anti-apoptotic effects of *Rhodiola sacra* were further evaluated in the brain tissues of CCI rats. Administration of *Rhodiola sacra* significantly reduced the number of apoptotic neuronal cells in the CA1 region. While the CCI group exhibited a high percentage of TUNEL-positive neurons (43.51%), indicating extensive apoptosis, the *Rhodiola sacra* treatment group showed a significantly lower percentage of TUNEL-positive neurons (20.47%) (Figs. **2A** and **C**). Additionally, *Rhodiola sacra* treatment decreased the expression of cleaved caspase-3 and Bax while increasing the expression of Bcl-2 compared to the CCI group (Fig. **2D**).

To elucidate the cellular and molecular mechanisms underlying mitochondrial damage in CCI, we examined the morphology of neuronal mitochondria in the rat hippocampus. Compared to sham-operated rats, hippocampal neurons in the CCI group exhibited altered mitochondrial morphology, characterized by reduced mitochondrial numbers and increased mitochondrial impairment (swollen, disorganized cristae). Importantly, *Rhodiola sacra* treatment significantly increased mitochondrial content in hippocampal neurons and markedly attenuated mitochondrial damage (Figs. **2E** and **F**, Fig. **S2**). Collectively, these results demonstrate that *Rhodiola sacra* effectively protects hippocampal neurons from CCI-induced apoptosis and promotes mitochondrial biogenesis, highlighting its potential as a therapeutic agent for chronic cerebral ischemia.

3.3. Salidroside Alleviates Neuronal Apoptosis against OGD Injury

To confirm that salidroside prevents hippocampal neuronal apoptosis against OGD injury, SH-SY5Y cells were pretreated with various concentrations of salidroside for 24 hours, followed by exposure to OGD for different durations (1, 4, 8, and 12 hours) and subsequent reculture under normoxic conditions. Initially, we determined that 4 hours of OGD treatment was optimal for subsequent experiments, as it resulted in approximately 50% cell viability (Fig. **3A**, Fig. **S3**). Under these conditions, salidroside significantly enhanced cell viability in a concentration-dependent manner, with the most pronounced effect observed at 100 μg/ml. Notably, further increasing the concentration to 200 μg/ml did not provide additional protection (Fig. **3B** and **C**). Next, we assessed neuronal apoptosis using flow cytometry and Western blot analysis. OGD treatment significantly increased the percentage of apoptotic cells, an effect that was markedly attenuated by pretreatment with salidroside at 50 μg/ml and 100 μg/ml (Fig. **3D** and **E**, Fig. **S4**). Additionally, OGD treatment promoted the activation of cleaved caspase-3 and Bax, while reducing Bcl-2 expression. These changes were effectively reversed by salidroside at 50 μg/ml and 100 μg/ml (Fig. **3F**, Fig. **S5**). Collectively, these findings demonstrate that salidroside exerts a significant protective effect against OGD-induced cell injury and apoptosis, highlighting its potential as a therapeutic agent for ischemic neuronal damage.

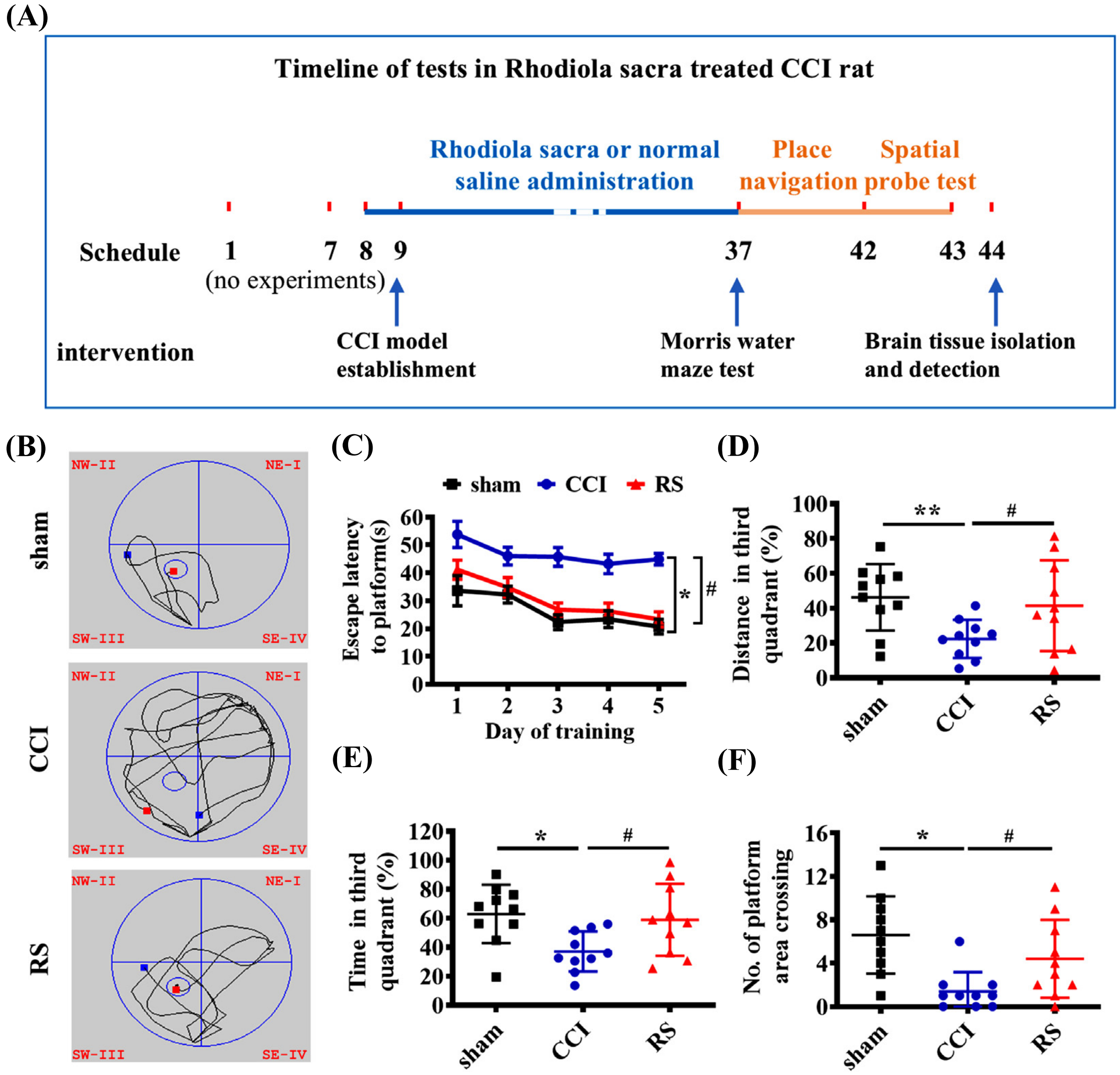

Fig. (1). *Rhodiola sacra* improves spatial learning and memory in CCI rats. In the water maze test, all rats were trained with a hidden platform for 5 days followed by a probe test without the platform 24 hours after the last training. There were significant effects of escape latencies among the three groups (n=10 rats per group) during acquisition training (two-way repeated-measures ANOVA: group, $F_{3,36}$=277.302, **p*<0.05; day, $F_{4,33}$=130.832, **p*<0.05). (**A**) Timeline of tests in *Rhodiola sacra* treated CCI rats. (**B**) The representative set of swimming track from rats undergoing cerebral ischemic injury with or without *Rhodiola sacra*. (**C**) Escape latency from rats undergoing cerebral ischemic injury with or without *Rhodiola sacra* during training trials. (**D**) Percentage of distance in target quadrant from rats undergoing cerebral ischemic injury with or without *Rhodiola sacra* during the probe test. (**E**) Percentage of time in target quadrant from rats undergoing cerebral ischemic injury with or without *Rhodiola sacra* during the probe test. (**F**) Number of platform area crossings from rats undergoing cerebral ischemic injury with or without *Rhodiola sacra* during the probe test. **p*<0.05 compared with sham group, ***p*<0.01 compared with sham group, #*p*<0.05 compared with CCI rats, One-way ANOVA. Data presented as mean ± SD. Scatter grams show individual values. (*A higher resolution/colour version of this figure is available in the electronic copy of the article*).

3.4. Salidroside Enhances Neuronal Mitochondrial Function against OGD Injury

To investigate whether salidroside mitigates hippocampal neuronal mitochondrial damage induced by OGD treatment, we assessed mitochondrial quality by evaluating oxidative stress levels, energy metabolism, and mitochondrial status. OGD treatment significantly elevated intracellular reactive oxygen species (ROS) and malondialdehyde (MDA) levels, while reducing superoxide dismutase (SOD) levels. Pretreatment with salidroside at concentrations of 50 μg/ml and 100 μg/ml effectively reversed these changes (Figs. **4A-C**). Additionally, OGD treatment increased the opening of the mitochondrial permeability transition pore (mPTP), resulting in decreased red fluorescence within mitochondria, as well as reduced mitochondrial membrane potential (MMP) and intracellular ATP levels. These OGD-induced damages were restored by salidroside at concentrations of 50 μg/ml and 100 μg/ml (Figs. **4D-G**). Collectively, these findings demonstrate that salidroside promotes mitochondrial function and protects against OGD-induced insults.

3.5. Salidroside Attenuates Neuronal Apoptosis and Promotes Mitochondrial Biogenesis *via* SIRT1 Upregulation

Previous studies have demonstrated that SIRT1 activation protects the brain against oxidative stress [56]. To investigate whether SIRT1 activation is involved in the protective effects of salidroside against oxygen-glucose deprivation

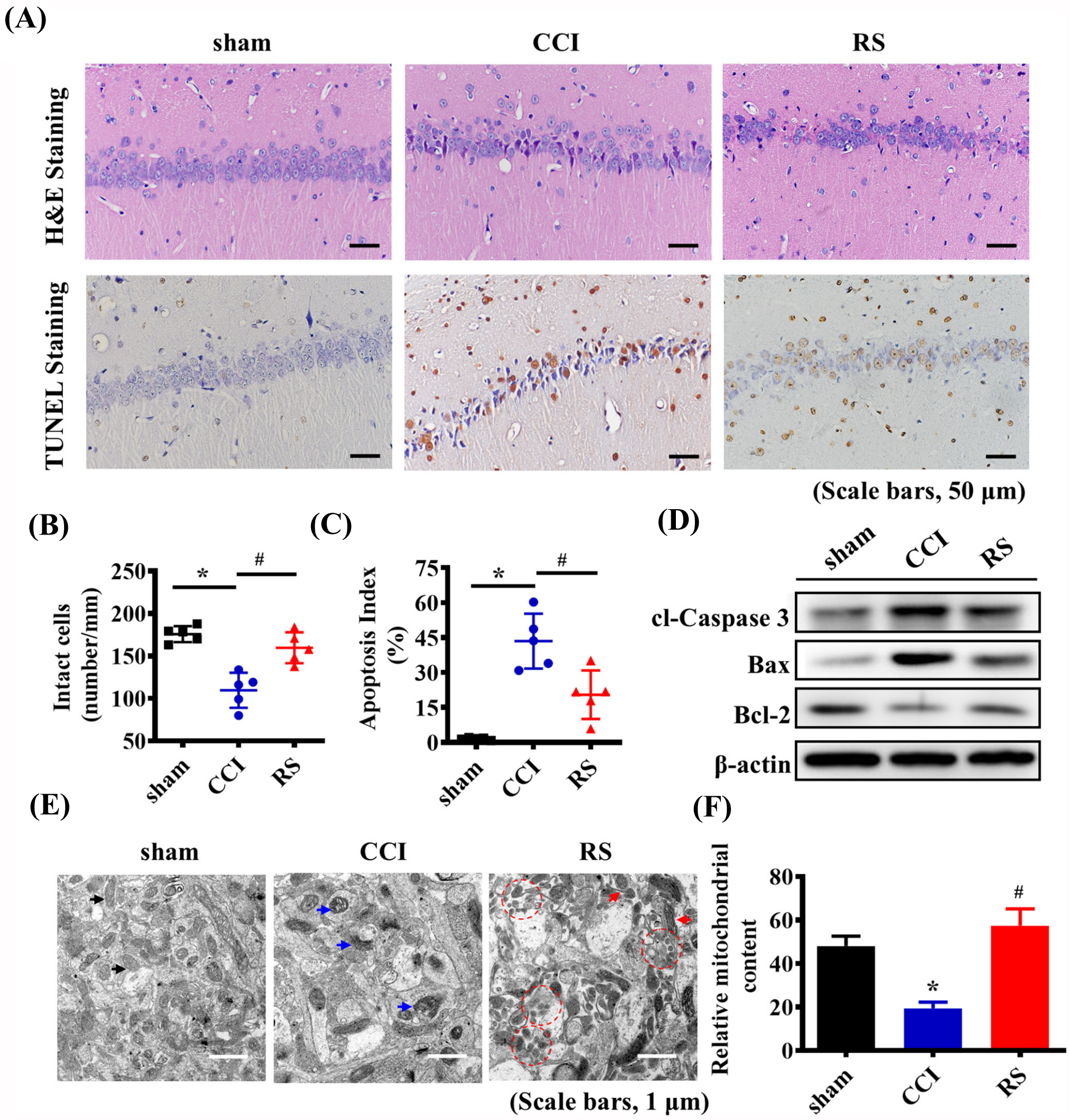

Fig. (2). *Rhodiola sacra* rescues ischemic brain injury and mitochondrial content in CCI rats. (**A**) Representative photos of hippocampal CA1 region of rats undergoing cerebral ischemic injury with or without *Rhodiola sacra* stained with HE and TUNEL, which showing more damaged and apoptosis cells in CCI rats than sham group, which can be reversed by *Rhodiola sacra*. (**B**) Intact neuronal cells of hippocampal CA1 area were obviously enhanced in *Rhodiola sacra* treatment group than CCI rats (n=5 rats). (**C**) Less TUNEL positive neuronal cells of hippocampal CA1 area were observed in *Rhodiola sacra* treatment group than CCI rats (n=5 rats). (**D**) Expressions of cleaved caspase 3, Bax and Bcl-2 were in hippocampus tissue analyzed by Western blotting. A representative blot is shown. (**E**) Representative electron micrographs of cerebral ischemic-injured rats with or without *Rhodiola sacra* treatment (n=5 rats). Black arrows: healthy mitochondria; blue arrows: damaged mitochondria; red arrows: recovering mitochondria; red encircled areas: newly formed mitochondria. (**F**) Quantification of mitochondrial content from electron microscopy images in rat hippocampus (n=5 rats). *$p<0.05$ compared with sham group, #$p<0.05$ compared with CCI rats, One-way ANOVA. Data presented as mean ± SD. Scatter grams show individual values. (*A higher resolution/colour version of this figure is available in the electronic copy of the article*).

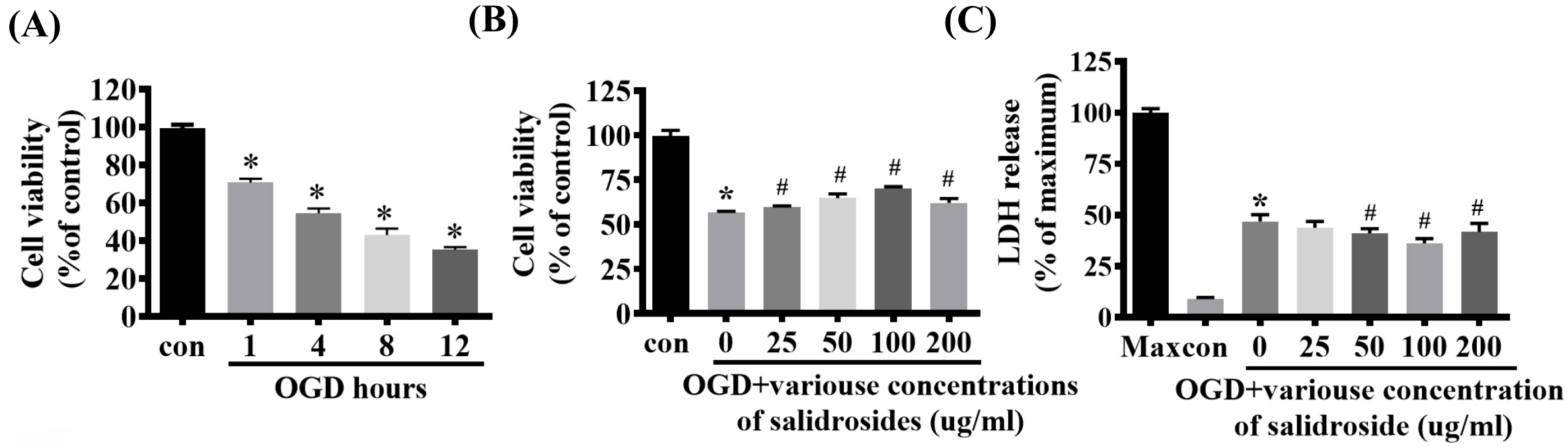

(Fig. 3) Contd….

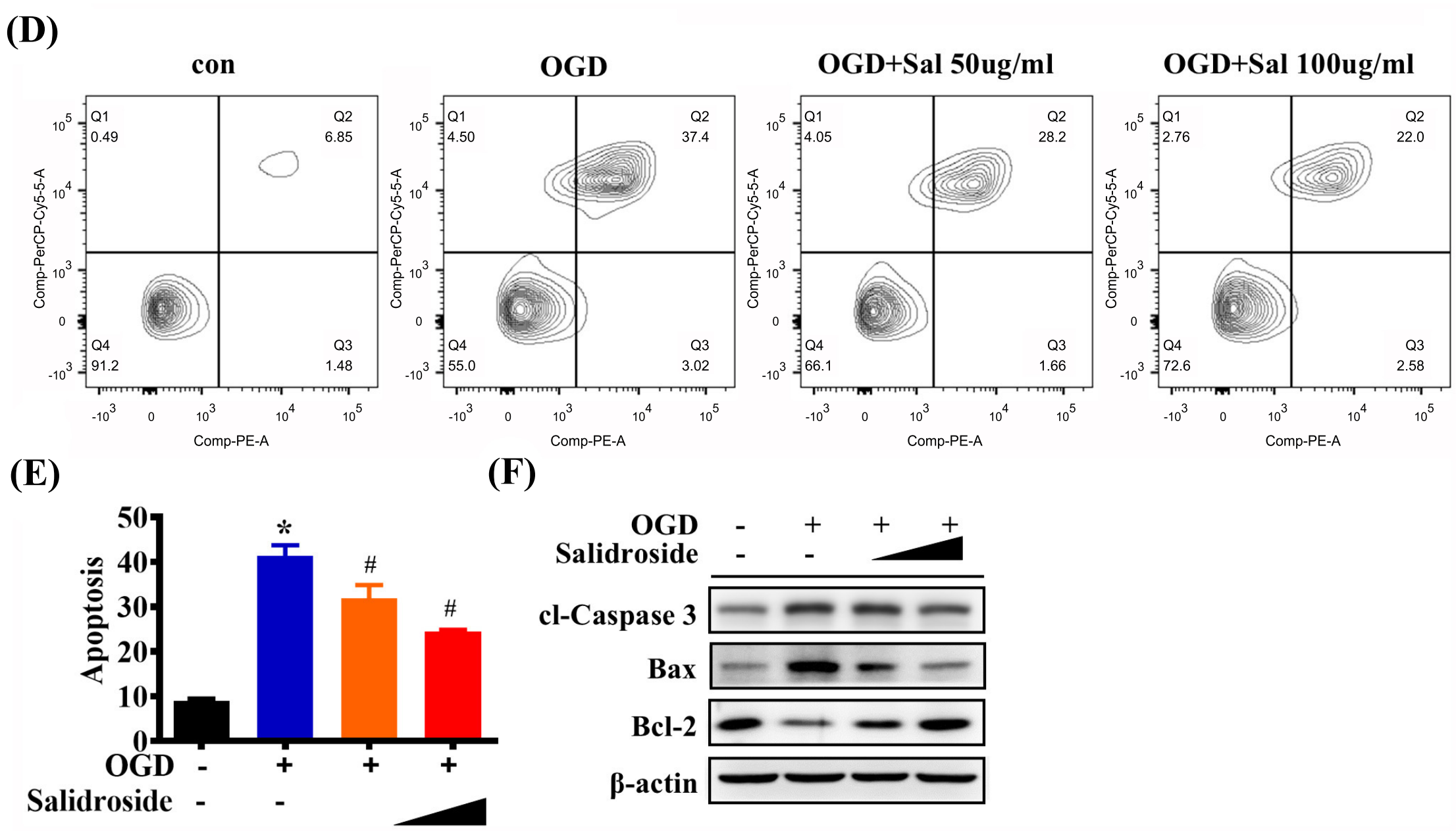

Fig. (3). Protective effects of salidroside on SH-SY5Y cell apoptosis induced by OGD injury. Cells were subjected to OGD for 4 hours with or without salidroside. (**A-C**) Cell viability was evaluated by MTS (**A** and **B**) and LDH assay (**C**). (**D-E**) Cells apoptosis was analyzed by flow cytometry. Representative graph of cell apoptosis (**D**). Quantification of apoptosis in SH-SY5Y cell (**E**). (**F**) Cleaved caspase 3, Bax and Bcl-2 were determined by Western blotting. A representative blot is shown. *p<0.05 compared with control cell, #p<0.05 compared with OGD group, One-way ANOVA. Each bar represents mean ± SD of at least 3 independent experiments preformed in triplicate. (*A higher resolution/colour version of this figure is available in the electronic copy of the article*).

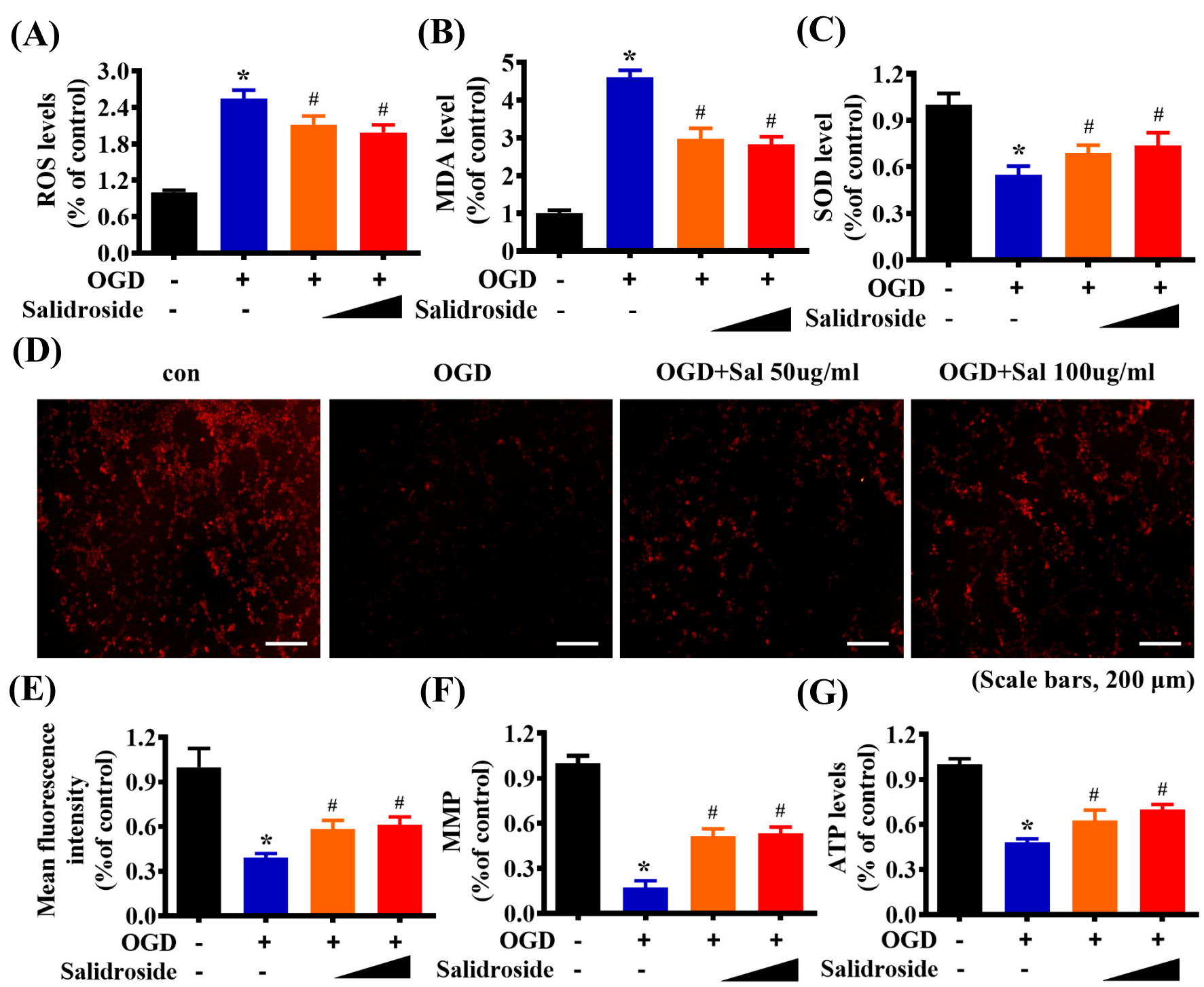

Fig. (4). Protective effects of salidroside on neuronal mitochondrial damage *in vitro*. Cells were subjected to OGD for 4 hours with or without salidroside. (**A-C**) The intracellular ROS levels (**A**) were analyzed by flow cytometer, the MDA levels (**B**) and SOD levels (**C**) were detected by respective commercial kits. (**D-E**) The mPTP was observed under a fluorescence microscope (**D**). Quantification of mitochondrial permeability transition pore (**E**) in SH-SY5Y cell. (**F**) The MMP was analyzed by flow cytometer. (**G**) The ATP levels were analyzed by flow cytometer. *p<0.05 compared with control group, #p<0.05 compared with OGD group, One-way ANOVA. Each bar represents mean ± SD of at least 3 independent experiments preformed in triplicate. (*A higher resolution/colour version of this figure is available in the electronic copy of the article*).

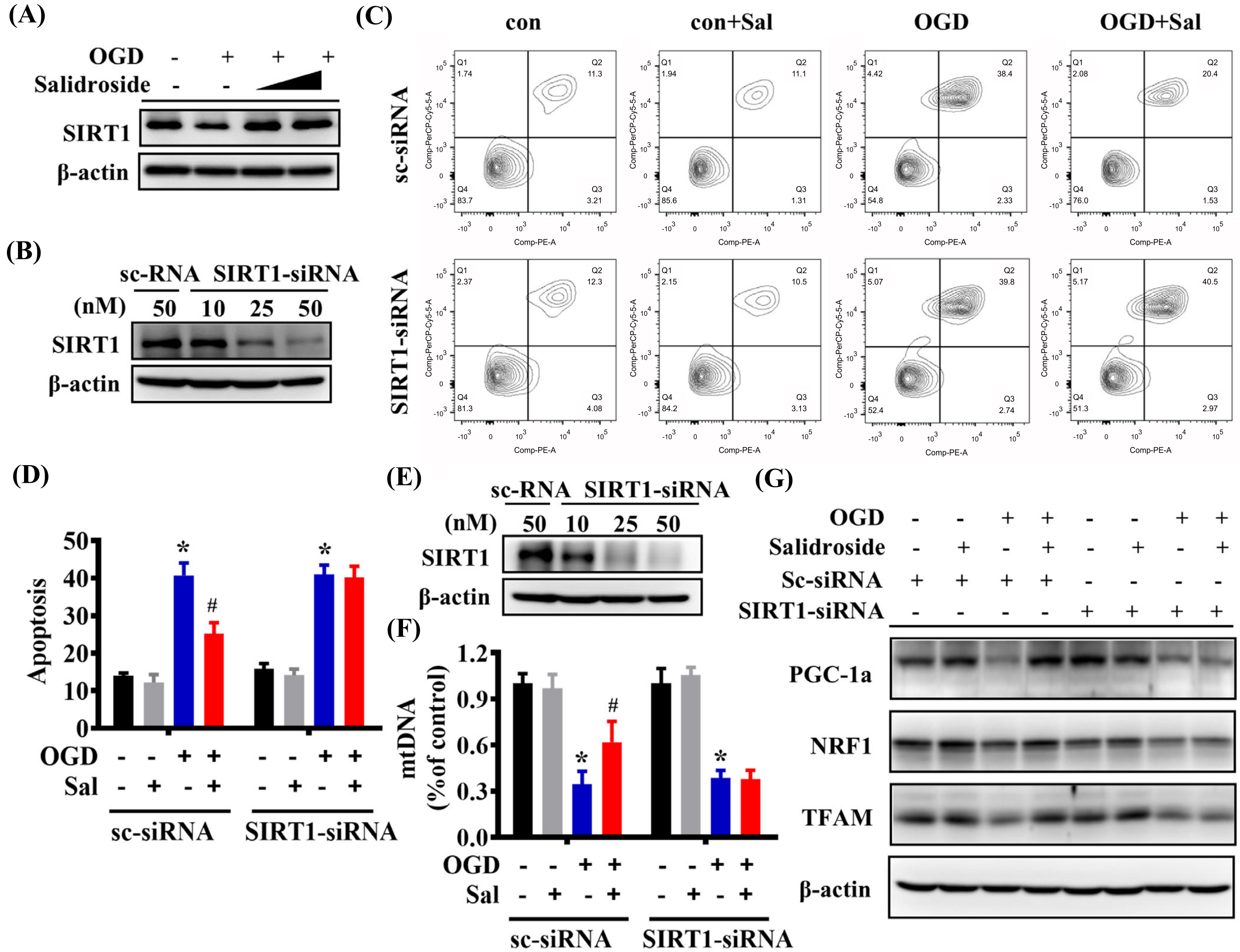

Fig. (5). Salidroside decreases SH-SY5Y apoptosis and promotes mitochondrial biogenesis against OGD injury *via* upregulation of SIRT1. (**A**) Cells were subjected to OGD for 4 hours with or without salidroside, protein levels of SIRT1 were measured. (**B**) Determination of SIRT1-siRNA knockdown efficiency by Western blotting. A representative blot is shown. (**C**, **D**) Cells transfected with SIRT1 siRNA or scrambled control siRNA were subjected to OGD for 4 hours with or without salidroside, and apoptosis was analyzed by flow cytometry. Representative graph of cell apoptosis (**C**). Quantification of apoptosis in SH-SY5Y cell (**D**). (**E**) Determination of SIRT1-siRNA knockdown efficiency by Western blotting. A representative blot is shown. (**F-G**) Cells transfected with SIRT1 siRNA or scrambled control siRNA were subjected to OGD for 4 hours with or without salidroside. The mtDNA was analyzed by qPCR assay (**F**), and PGC-1a, NRF1 and TFAM expression was analyzed by Western blotting (**G**). A representative blot is shown. **p*<0.05 compared with control group, #*p*<0.05 compared with OGD group, One-way ANOVA. Each bar represents mean ± SD of at least 3 independent experiments preformed in triplicate. (*A higher resolution/colour version of this figure is available in the electronic copy of the article*).

(OGD) injury, we first examined SIRT1 expression in SH-SY5Y cells. OGD treatment significantly downregulated SIRT1 expression, but this reduction was reversed considerably by salidroside at concentrations of 50 μg/ml and 100 μg/ml (Fig. **5A**). To determine whether the anti-apoptotic effects of salidroside are mediated by SIRT1 activation, SH-SY5Y cells were transfected with SIRT1 siRNA or scrambled control siRNA (scRNA) and then subjected to OGD injury after 24 hours of salidroside pretreatment. SIRT1 siRNA effectively suppressed SIRT1 expression in a concentration-dependent manner (Fig. **5B**). The anti-apoptotic effects of salidroside were abolished by SIRT1 inhibition (Figs. **5C** and **D**), indicating that the anti-apoptotic actions of salidroside against OGD injury are dependent on SIRT1 activation.

Given that SIRT1 regulates the transcription of PGC-1α and other mitochondrial-related genes, we explored whether salidroside-mediated mitochondrial protection is mediated by SIRT1-driven mitochondrial biogenesis. We measured the levels of key regulators of mitochondrial biogenesis, including PGC-1α, nuclear respiratory factor 1 (NRF1), mitochondrial transcription factor A (TFAM), and mtDNA content. OGD treatment decreased mtDNA content in control cells, an effect that was significantly reversed by salidroside. However, this reversal was abolished by SIRT1 inhibition (Fig. **5E** and **F**). Additionally, OGD treatment suppressed PGC-1α expression, which was restored by salidroside at 100 μg/ml. SIRT1 siRNA abolished this restoration. Similarly, reductions in NRF1 and TFAM protein levels following OGD injury were attenuated by salidroside, an effect that

was abolished by SIRT1 inhibition (Fig. **5G**). These findings suggest that salidroside prevents OGD-induced suppression of mitochondrial biogenesis by activating SIRT1.

3.6. *Rhodiola sacra* Promotes SIRT1-mediated Mitochondrial Biogenesis in CCI Rats

To further investigate the role of SIRT1 in the neuroprotective effects of *Rhodiola sacra*, we examined SIRT1 expression in the brain tissues of CCI rats. SIRT1 mRNA expression was significantly reduced in CCI rats compared to the sham group, but this reduction was significantly reversed by *Rhodiola sacra* treatment (Fig. **6A**). The protein levels of SIRT1 were consistent with the mRNA expression levels (Fig. **6B**). We also investigated whether PGC-1α is involved in the neuroprotective effects of *Rhodiola sacra* against chronic ischemic cerebral damage. The mRNA expression levels of PGC-1α, NRF1, and TFAM were significantly decreased in CCI rats, but these reductions were reversed by *Rhodiola sacra* treatment (Fig. **6C**). The protein levels of PGC-1α, NRF1, and TFAM were consistent with their mRNA expression levels (Fig. **6D**). Collectively, these results indicate that *Rhodiola sacra* protects against CCI-induced reductions in SIRT1 expression and promotes mitochondrial biogenesis, suggesting that the SIRT1/PGC-1α pathway is involved in the neuroprotective effects of *Rhodiola sacra*.

4. DISCUSSION

CCI triggers a cascade of hippocampal neuronal dysfunction characterized by oxidative stress, mitochondrial impairment, and apoptotic signaling, ultimately leading to cognitive decline and dementia, a growing public health concern [57-62]. While current clinical interventions such as thrombolysis and mechanical thrombectomy effectively restore acute cerebral perfusion, these strategies fail to address the persistent mitochondrial dysfunction and progressive neuronal damage underlying chronic cognitive deficits [63-65]. Consequently, pharmacological strategies targeting these chronic pathological sequelae have garnered increasing attention [66]. In this study, we demonstrate that RS and its active component salidroside attenuate CCI-induced hippocampal neuronal apoptosis by activating mitochondrial biogenesis through the SIRT1/PGC-1α pathway. These findings position RS and salidroside as promising candidates for mitigating chronic ischemic injury, providing a mechanistic foundation for their clinical translation.

In this investigation, chronic cerebral ischemia was modelled *via* permanent bilateral typical carotid artery occlusion. *Rhodiola sacra* administration markedly restored spatial learning and memory, an effect accompanied by reduced neuronal apoptosis and increased mitochondrial density within the hippocampus. In parallel, salidroside, the principal bioactive constituent of *Rhodiola sacra*, rescued mitochondrial function and suppressed apoptosis in SH-SY5Y cells subjected to oxygen-glucose deprivation. Notably, while AMPK activation has been implicated as an upstream regulator of SIRT1 in other ischemia models [67, 68], our data revealed that salidroside's neuroprotective effects were independent of AMPK signaling. Total AMPK expression remained unchanged, and OGD-induced P-AMPK elevation was not further modulated by salidroside (Fig. **S6**). This suggests that RS directly targets SIRT1, bypassing the canonical AMPK-dependent activation pathway. Critically, genetic silencing of SIRT1 abolished these protective effects, establishing the SIRT1/PGC-1α-mediated mitochondrial biogenesis axis as indispensable for *Rhodiola sacra*-induced neuroprotection. These data corroborate prior evidence positioning SIRT1 as a master regulator of mitochondrial biogenesis [22] and uniquely identify *Rhodiola sacra* as a naturally occurring SIRT1 activator with substantial therapeutic potential for ischemic brain injury.

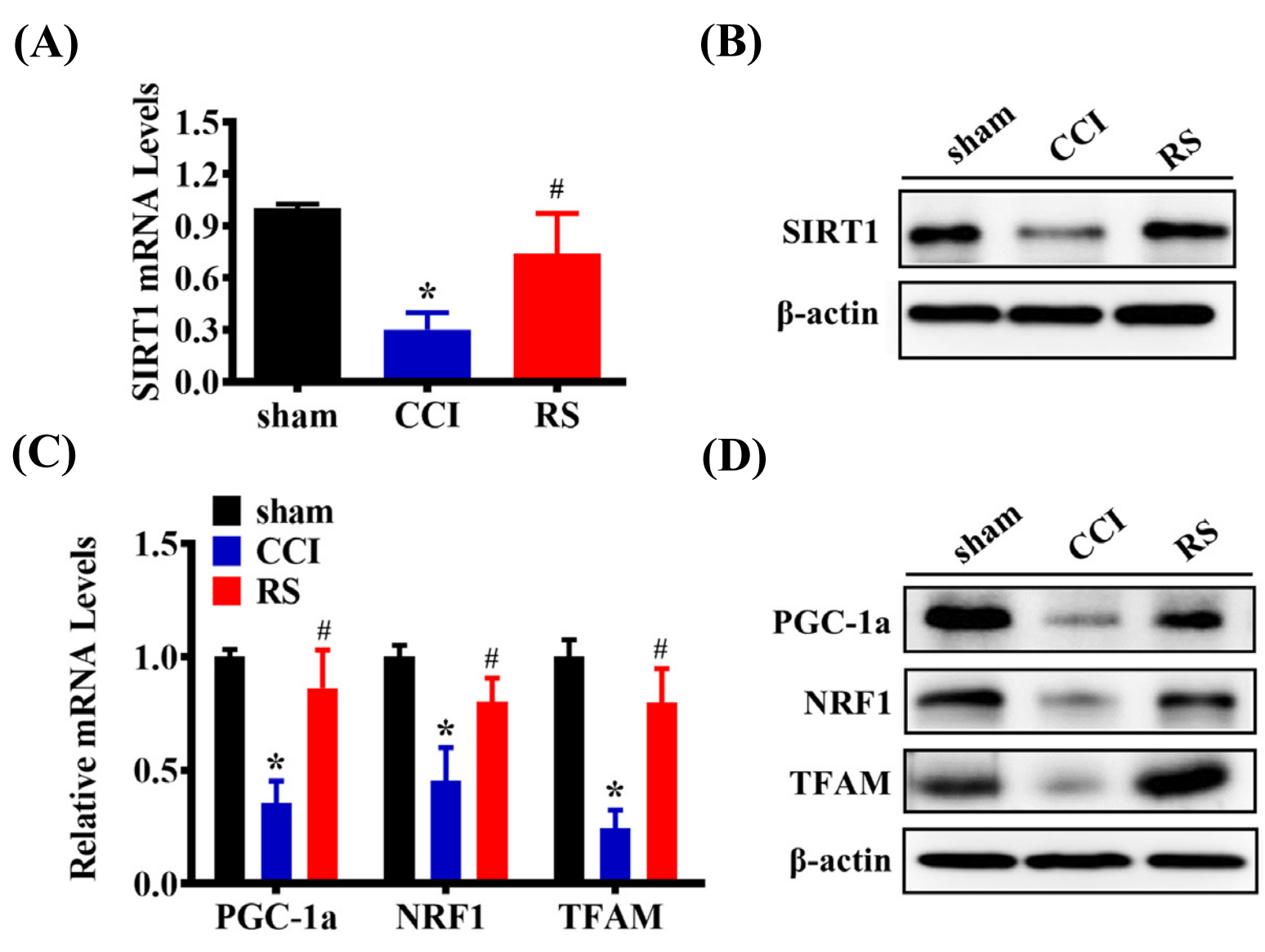

Fig. (6). SIRT1-Mediated Mitochondrial Biogenesis Promoted by Rhodiola rosea in CCI rats. (**A**, **B**) Expression of SIRT1 in hippocampus tissue were analyzed at transcription level (**A**) and protein level (**B**). A representative blot is shown. (**C**, **D**) Expression of PGC-1a, NRF1 and TFAM in hippocampus tissue were determined at transcription level (**C**) and protein level (**D**). A representative blot is shown. *p<0.05 compared with control group, #p<0.05 compared with OGD group, One-way ANOVA. Each bar represents mean ± SD of at least 3 independent experiments preformed in triplicate. (*A higher resolution/colour version of this figure is available in the electronic copy of the article*).

The central role of the SIRT1/PGC-1α axis in this neuroprotective mechanism is further underscored by its ability to link RS/salidroside-mediated effects to CCI pathology. Mitochondrial biogenesis, orchestrated by this pathway, serves as a critical reparative response in CCI, and its impairment exacerbates oxidative stress and neuronal apoptosis [3, 69-72]. Notably, our comprehensive assessment of redox regulators (Nrf2/HO-1), inflammatory mediators (NF-κB, IL-1β, and IL-6), and metabolic sensors (PPAR-γ/α) revealed no alterations following salidroside treatment (Fig. **S7**). This compelling absence of effect confirms that SIRT1-driven MB operates as the dominant pathway, functioning independently of antioxidant defenses, cytokine-driven inflammation, or nuclear receptor signaling. Although UCP2, a downstream effector of PGC-1α, has been reported to mitigate oxidative stress in other models [73, 74], we observed that salidroside's antioxidant effects occurred without UCP2 upregulation (Fig. **S6**). This implies that alternative antioxidant mechanisms (*e.g.*, SOD induction) may compensate for UCP2's absence, highlighting the adaptability of the SIRT1/PGC-1α pathway in different pathological contexts.

SIRT1 operates as a transcriptional checkpoint that governs this process; its activation mitigates ischemic injury and has been advanced as a putative therapeutic target [75-77]. Notably, emerging evidence indicates that SIRT1 confers neuroprotection by negatively regulating the Notch1/Hes1 axis *via* a deacetylation-dependent mechanism [78]. Moreover, the Notch1/Hes1 axis has been reported to suppress PGC-1α-mediated mitochondrial biogenesis in chronic kidney disease [79]. Against this backdrop, we demonstrate that salidroside up-regulates SIRT1 expression, thereby activating PGC-1α and downstream regulators of MB (NRF1, TFAM), with consequent restoration of mitochondrial mass and integrity. Critically, SIRT1 knockdown abolishes these salidroside-induced improvements, confirming the indispensability role of the SIRT1/PGC-1α pathway in both mitochondrial biogenesis and anti-apoptotic effects.

Conventional neuroprotectants such as edaravone, whose efficacy is limited to single-target antioxidant scavenging, fail to engage these restorative mitochondrial programs and thus leave the underlying bioenergetic deficit unaddressed [80, 81]. By contrast, *Rhodiola sacra* and its bioactive constituent salidroside instead target the etiological core of chronic neuronal dysfunction by orchestrating SIRT1-dependent MB; they simultaneously rectify mitochondrial failure and attenuate apoptosis, offering a mechanistically grounded and clinically translatable strategy for mitigating post-ischemic cognitive impairment.

Although this study offers convincing evidence for the therapeutic role of RS in treating CCI, several important issues warrant further consideration. Sexual dimorphism plays a significant role in influencing ischemic stroke outcomes, with hormonal and genetic factors modulating neuroprotection [82]. However, the present study predominantly focuses on male mice and lacks in-depth research on the intervention effects and mechanisms of RS in female mice. As emphasized by Bushnell *et al*., neglecting sex differences may lead to biased therapeutic predictions [83]. Future research will address this gap by evaluating the effects of RS in female rodents and incorporating hormone-synchronized models. Additionally, the administration of RS in this study began prior to CCI induction, which deviates from clinical practice, where neuroprotection is typically initiated post-insult. This pre-injury administration design limits the direct extrapolation of the findings to salvage therapy in established ischemia. Future studies should assess the efficacy of RS in post-ischemia treatment windows that align with clinical guidelines to enhance clinical relevance.

CONCLUSION

In this study, we elucidate a novel mechanism by which *Rhodiola sacra* protects against CCI-induced injury. Specifically, *Rhodiola sacra* mitigates chronic ischemic brain damage by activating SIRT1, which in turn upregulates PGC-1α and promotes mitochondrial biogenesis, independently of AMPK-mediated oxidative stress, inflammatory or metabolic reprogramming pathways. Thus, the SIRT1/PGC-1α pathway may represent a promising therapeutic target for cerebral ischemic injury, and *Rhodiola sacra* could serve as a potential therapeutic agent to slow the progression of chronic cerebral ischemia.

AUTHORS' CONTRIBUTIONS

The authors confirm contribution to the paper as follows: study conception and design: L.Y.Z, S.W., Z.F, and Z.Y; data collection: L.Y.Z, J.J, and J.P.L.; analysis and interpretation of results: L.Y.Z., and S.W.; draft manuscript: L.Y.Z., and S.W. All authors reviewed the results and approved the final version of the manuscript.

LIST OF ABBREVIATIONS

CCI	=	Chronic Cerebral Ischemia
MB	=	Mitochondrial Biogenesis
MMP	=	Mitochondrial Membrane Potential
mPTP	=	Mitochondrial Permeability Transition Pore
mtDNA	=	Mitochondrial DNA
OGD	=	Oxygen-glucose Deprivation
ROS	=	Reactive Oxygen Species
RS	=	*Rhodiola Sacra*
siRNA	=	Small Interfering RNA

ETHICS APPROVAL AND CONSENT TO PARTICIPATE

All experimental studies involving animals were reviewed and approved by the Institutional Animal Care and Use Committee of Tianjin Medical University, Tianjin, China. The associated approval number is TMUaMEC 2024056.

HUMAN AND ANIMAL RIGHTS

This study adheres to internationally accepted standards for animal research and follows the 3Rs principle. The ARRIVE guidelines were employed for reporting experiments involving live animals, promoting ethical research practices.

The animal experimentation was conducted in accordance with the US National Research Council's "Guide for the Care and Use of Laboratory Animals".

CONSENT FOR PUBLICATION

Not applicable.

AVAILABILITY OF DATA AND MATERIALS

All the data and supporting information are provided within the article.

FUNDING

None.

CONFLICT OF INTEREST

The authors declare no conflict of interest, financial or otherwise.

ACKNOWLEDGEMENTS

Declared none.

SUPPLEMENTARY MATERIAL

Supplementary material is available on the publisher's website along with the published article.

REFERENCES

[1] Zhao, Y.; Zhang, X.; Chen, X.; Wei, Y. Neuronal injuries in cerebral infarction and ischemic stroke: From mechanisms to treatment (Review). *Int. J. Mol. Med.,* **2021**, *49*(2), 15. http://dx.doi.org/10.3892/ijmm.2021.5070 PMID: 34878154

[2] Sadeghzadeh, J.; Hosseini, L.; Mobed, A.; Zangbar, H.S.; Jafarzadeh, J.; Pasban, J.; Shahabi, P. The impact of cerebral ischemia on antioxidant enzymes activity and neuronal damage in the hippocampus. *Cell. Mol. Neurobiol.,* **2023**, *43*(8), 3915-3928. http://dx.doi.org/10.1007/s10571-023-01413-w PMID: 37740074

[3] Lan, X.; Wang, Q.; Liu, Y.; You, Q.; Wei, W.; Zhu, C.; Hai, D.; Cai, Z.; Yu, J.; Zhang, J.; Liu, N. Isoliquiritigenin alleviates cerebral ischemia-reperfusion injury by reducing oxidative stress and ameliorating mitochondrial dysfunction *via* activating the Nrf2 pathway. *Redox Biol.,* **2024**, *77*, 103406. http://dx.doi.org/10.1016/j.redox.2024.103406 PMID: 39454290

[4] Yuan, Y.; Tian, Y.; Jiang, H.; Cai, L.; Song, J.; Peng, R.; Zhang, X. Mechanism of PGC-1α-mediated mitochondrial biogenesis in cerebral ischemia–reperfusion injury. *Front. Mol. Neurosci.,* **2023**, *16*, 1224964. http://dx.doi.org/10.3389/fnmol.2023.1224964 PMID: 37492523

[5] Frank, D.; Zlotnik, A.; Boyko, M.; Gruenbaum, B.F. The development of novel drug treatments for stroke patients: A review. *Int. J. Mol. Sci.,* **2022**, *23*(10), 5796. http://dx.doi.org/10.3390/ijms23105796 PMID: 35628606

[6] Seiffge, D.J. Intravenous thrombolytic therapy for treatment of acute ischemic stroke in patients taking non–vitamin k antagonist oral anticoagulants. *JAMA,* **2022**, *327*(8), 725-726. http://dx.doi.org/10.1001/jama.2022.0068 PMID: 35143602

[7] Jia, M.; Miao, W.; Li, Y.; Guo, Y.; Zeng, J.; Gao, Y.; Li, X.; Wang, Y.; Zhao, Z.; Xu, Z.; Wang, N.; Yang, F.; Zhao, J.; Wang, Y.; Wang, G. A polymerized probucol nanoformulation with neutrophil extracellular vesicle camouflage for cerebral ischemia-reperfusion injury therapy. *Innovation (Camb),* **2025**, *6*(4), 100761. http://dx.doi.org/10.1016/j.xinn.2024.100761 PMID: 40470331

[8] Wu, F.; Lai, S.; Fu, D.; Liu, J.; Wang, C.; Feng, H.; Liu, J.; Li, Z.; Li, P. Neuroprotective effects and metabolomics study of protopanaxatriol (PPT) on cerebral ischemia/reperfusion injury *in vitro* and *in vivo. Int. J. Mol. Sci.,* **2023**, *24*(2), 1789. http://dx.doi.org/10.3390/ijms24021789 PMID: 36675303

[9] Yu, X.; Wei, P.; Chen, Z.; Li, X.; Zhang, W.; Yang, Y.; Liu, C.; Zhao, S.; Li, X.; Liu, X. Comparative analysis of the organelle genomes of three Rhodiola species provide insights into their structural dynamics and sequence divergences. *BMC Plant. Biol.,* **2023**, *23*(1), 156. http://dx.doi.org/10.1186/s12870-023-04159-1 PMID: 36944988

[10] Jówko, E.; Sadowski, J.; Długołęcka, B.; Gierczuk, D.; Opaszowski, B.; Cieśliński, I. Effects of *Rhodiola rosea* supplementation on mental performance, physical capacity, and oxidative stress biomarkers in healthy men. *J. Sport. Health. Sci.,* **2018**, *7*(4), 473-480. http://dx.doi.org/10.1016/j.jshs.2016.05.005 PMID: 30450257

[11] Xing, N.; Qin, J.; Ren, D.; Du, Q.; Li, Y.; Mi, J.; Zhang, F.; Ai, L.; Zhang, S.; Zhang, Y.; Wang, S. Integrating UPLC-Q-Exactive Orbitrap/MS, network pharmacology and experimental validation to reveal the potential mechanism of Tibetan medicine Rhodiola granules in improving myocardial ischemia-reperfusion injury. *J. Ethnopharmacol.,* **2023**, *314*, 116572. http://dx.doi.org/10.1016/j.jep.2023.116572 PMID: 37201662

[12] Man, C.; Dai, Z.; Fan, Y. Dazhu Hongjingtian preparation as adjuvant therapy for unstable angina pectoris: A meta-analysis of randomized controlled trials. *Front. Pharmacol.,* **2020**, *11*, 213. http://dx.doi.org/10.3389/fphar.2020.00213 PMID: 32210811

[13] Zheng, X.; Zhang, H.; Zhang, Y.; Ding, Z.; Huang, Z.; Li, H.; Yao, M.; Song, W.; Liu, J. Salidroside ameliorates cerebral ischemic injury and regulates the glutamate metabolism pathway in astrocytes. *Front. Pharmacol.,* **2024**, *15*, 1472100. http://dx.doi.org/10.3389/fphar.2024.1472100 PMID: 39600364

[14] Dun, Y.; Liu, S.; Zhang, W.; Xie, M.; Qiu, L. Exercise combined with *Rhodiola sacra* supplementation improves exercise capacity and ameliorates exhaustive exercise-induced muscle damage through enhancement of mitochondrial quality control. *Oxid. Med. Cell. Longev.,* **2017**, *2017*(1), 8024857. http://dx.doi.org/10.1155/2017/8024857 PMID: 29359009

[15] Zhang, X.; Zhang, H.; Liu, Z.; Huang, T.; Yi, R.; Ma, Z.; Gao, Y. Salidroside improves blood–brain barrier integrity and cognitive function in hypobaric hypoxia mice by inhibiting microglia activation through GSK3β. *Phytother. Res.,* **2025**, *39*(4), 1808-1825. http://dx.doi.org/10.1002/ptr.8264 PMID: 39364585

[16] Lu, S.; Ji, N.; Wang, W.; Lin, X.; Gao, D.; Geng, D. Salidroside improves cognitive function in Parkinson's disease *via* Braf-mediated mitogen-activated protein kinase signaling pathway. *Biomed. Pharmacother.,* **2024**, *177*, 116968. http://dx.doi.org/10.1016/j.biopha.2024.116968 PMID: 38901199

[17] Yang, S.; xie, Z.; Pei, T.; zeng, Y.; Xiong, Q.; Wei, H.; Wang, Y.; Cheng, W. Salidroside attenuates neuronal ferroptosis by activating the Nrf2/HO1 signaling pathway in $A\beta_{1-42}$-induced Alzheimer's disease mice and glutamate-injured HT22 cells. *Chin Med.,* **2022**, *17*(1), 82. http://dx.doi.org/10.1186/s13020-022-00634-3 PMID: 35787281

[18] Lai, W.; He, Y.; Zhou, B.; Wu, Q.; Wu, H.; Chen, J.; Zheng, X.; Jia, R.; Lin, P.; Hong, G.; Chen, J. Salidroside facilitates neuroprotective effects in ischemic stroke by promoting axonal sprouting through promoting autophagy. *Phytomedicine,* **2024**, *135*, 156208. http://dx.doi.org/10.1016/j.phymed.2024.156208 PMID: 39550919

[19] Hou, Y.; Fan, F.; Xie, N.; Zhang, Y.; Wang, X.; Meng, X. Rhodiola crenulata alleviates hypobaric hypoxia-induced brain injury by maintaining BBB integrity and balancing energy metabolism dysfunction. *Phytomedicine,* **2024**, *128*, 155529. http://dx.doi.org/10.1016/j.phymed.2024.155529 PMID: 38503156

[20] Wen, B.; Zhou, K.; Hu, C.; Chen, J.; Xu, K.; Liang, T.; He, B.; Chen, L.; Chen, J. Salidroside Ameliorates ischemia-induced neuronal injury through AMPK dependent and independent pathways to maintain mitochondrial quality control. *Am. J. Chin Med.,* **2022**, *50*(4), 1133-1153. http://dx.doi.org/10.1142/S0192415X2250046X PMID: 35543160

[21] Ji, Z.; Liu, G.H.; Qu, J. Mitochondrial sirtuins, metabolism, and aging. *J. Genet. Genomics,* **2022**, *49*(4), 287-298. http://dx.doi.org/10.1016/j.jgg.2021.11.005 PMID: 34856390

[22] Luen Tang, B. Sirt1 and the mitochondria. *Mol. Cells,* **2016**, *39*(2), 87-95.

http://dx.doi.org/10.14348/molcells.2016.2318 PMID: 26831453

[23] Liu, Y.; Yang, H.; Luo, N.; Fu, Y.; Qiu, F.; Pan, Z.; Li, X.; Jian, W.; Yang, X.; Xue, Q.; Luo, Y.; Yu, B.; Liu, Z. An Fgr kinase inhibitor attenuates sepsis-associated encephalopathy by ameliorating mitochondrial dysfunction, oxidative stress, and neuroinflammation *via* the SIRT1/PGC-1α signaling pathway. *J. Transl. Med.,* **2023**, *21*(1), 486. http://dx.doi.org/10.1186/s12967-023-04345-7 PMID: 37475042

[24] Yao, J.; Wang, J.; Xu, Y.; Guo, Q.; Sun, Y.; Liu, J.; Li, S.; Guo, Y.; Wei, L. CDK9 inhibition blocks the initiation of PINK1-PRKN-mediated mitophagy by regulating the SIRT1-FOXO3-BNIP3 axis and enhances the therapeutic effects involving mitochondrial dysfunction in hepatocellular carcinoma. *Autophagy,* **2022**, *18*(8), 1879-1897. http://dx.doi.org/10.1080/15548627.2021.2007027 PMID: 34890308

[25] Halling, J.F.; Pilegaard, H. PGC-1α-mediated regulation of mitochondrial function and physiological implications. *Appl. Physiol. Nutr. Metab.,* **2020**, *45*(9), 927-936. http://dx.doi.org/10.1139/apnm-2020-0005 PMID: 32516539

[26] Abu Shelbayeh, O.; Arroum, T.; Morris, S.; Busch, K.B. PGC-1α is a master regulator of mitochondrial lifecycle and ROS stress response. *Antioxidants,* **2023**, *12*(5), 1075. http://dx.doi.org/10.3390/antiox12051075 PMID: 37237941

[27] Zhao, Y.; Zhang, J.; Zheng, Y.; Zhang, Y.; Zhang, X.J.; Wang, H.; Du, Y.; Guan, J.; Wang, X.; Fu, J. NAD^+ improves cognitive function and reduces neuroinflammation by ameliorating mitochondrial damage and decreasing ROS production in chronic cerebral hypoperfusion models through Sirt1/PGC-1α pathway. *J. Neuroinflammation,* **2021**, *18*(1), 207. http://dx.doi.org/10.1186/s12974-021-02250-8 PMID: 34530866

[28] Yu, Y.; Zhao, Y.; Teng, F.; Li, J.; Guan, Y.; Xu, J.; Lv, X.; Guan, F.; Zhang, M.; Chen, L. Berberine improves cognitive deficiency and muscular dysfunction *via* activation of the AMPK/SIRT1/PGC-1a pathway in skeletal muscle from naturally aging rats. *J. Nutr. Health. Aging,* **2018**, *22*(6), 710-717. http://dx.doi.org/10.1007/s12603-018-1015-7 PMID: 29806860

[29] Bi, A.; Liu, R.; Xie, M.; He, B.; Yan, T.; Du, Y.; Jia, Y. Semen Ziziphi Spinosae alleviates cardiomyocyte apoptosis in rats with coronary heart disease *via* the AMPK/SIRT1/PGC-1alpha signaling pathway activation. *Phytomedicine,* **2025**, *142*, 156743. http://dx.doi.org/10.1016/j.phymed.2025.156743 PMID: 40250033

[30] Feng, X.; Zheng, Y.; Mao, N.; Shen, M.; Chu, L.; Fang, Y.; Pang, M.; Wang, Z.; Lin, Z. Menaquinone-4 alleviates hypoxic-ischemic brain damage in neonatal rats by reducing mitochondrial dysfunction *via* Sirt1-PGC-1α-TFAM signaling pathway. *Int. Immunopharmacol.,* **2024**, *134*, 112257. http://dx.doi.org/10.1016/j.intimp.2024.112257 PMID: 38759366

[31] Cao, Y.; Yao, W.; Yang, T.; Yang, M.; Liu, Z.; Luo, H.; Cao, Z.; Chang, R.; Cui, Z.; Zuo, H.; Liu, B. Elucidating the mechanisms of Buyang Huanwu Decoction in treating chronic cerebral ischemia: A combined approach using network pharmacology, molecular docking, and *in vivo* validation. *Phytomedicine,* **2024**, *132*, 155820. http://dx.doi.org/10.1016/j.phymed.2024.155820 PMID: 39004032

[32] Wang, J.; Li, Q.; Chu, S.; Liu, X.; Zhang, J.; He, W. Impact of Codonopsis decoction on cerebral blood flow and cognitive function in rats with chronic cerebral ischemia. *J. Ethnopharmacol.,* **2024**, *323*, 117585. http://dx.doi.org/10.1016/j.jep.2023.117585 PMID: 38159825

[33] Zhu, Z.; Tao, X.; Dai, T.; Wu, J.; Han, C.; Huang, P.; Gong, W. Cognitive-exercise dual-task attenuates chronic cerebral ischemia-induced cognitive impairment by activating cAMP/PKA pathway through inhibiting EphrinA3/EphA4. *Exp. Neurol.,* **2024**, *372*, 114617. http://dx.doi.org/10.1016/j.expneurol.2023.114617 PMID: 38007209

[34] Xu, J.; Zhang, L.; Li, M.; He, X.; Luo, J.; Wu, R.; Hong, Z.; Zheng, H.; Hu, X. TREM2 mediates physical exercise-promoted neural functional recovery in rats with ischemic stroke *via* microglia-promoted white matter repair. *J. Neuroinflammation,* **2023**, *20*(1), 50. http://dx.doi.org/10.1186/s12974-023-02741-w PMID: 36829205

[35] Zhou, H.; Yan, L.; Huang, H.; Li, X.; Xia, Q.; Zheng, L.; Shao, B.; Gao, Q.; Sun, N.; Shi, J. Tat-NTS peptide protects neurons against cerebral ischemia-reperfusion injury *via* ANXA1 SUMOylation in microglia. *Theranostics,* **2023**, *13*(15), 5561-5583. http://dx.doi.org/10.7150/thno.85390 PMID: 37908731

[36] Bai, S.; Lu, X.; Pan, Q.; Wang, B.; Pong U, K.; Yang, Y.; Wang, H.; Lin, S.; Feng, L.; Wang, Y.; Li, Y.; Lin, W.; Wang, Y.; Zhang, X.; Li, Y.; Li, L.; Yang, Z.; Wang, M.; Lee, W.Y.W.; Jiang, X.; Li, G. Cranial bone transport promotes angiogenesis, neurogenesis, and modulates meningeal lymphatic function in middle cerebral artery occlusion rats. *Stroke,* **2022**, *53*(4), 1373-1385. http://dx.doi.org/10.1161/STROKEAHA.121.037912 PMID: 35135326

[37] Fu, C.; Wu, Y.; Liu, S.; Luo, C.; Lu, Y.; Liu, M.; Wang, L.; Zhang, Y.; Liu, X. Rehmannioside A improves cognitive impairment and alleviates ferroptosis *via* activating PI3K/AKT/Nrf2 and SLC7A11/GPX4 signaling pathway after ischemia. *J. Ethnopharmacol.,* **2022**, *289*, 115021. http://dx.doi.org/10.1016/j.jep.2022.115021 PMID: 35091012

[38] Xu, J.; Ji, T.; Li, G.; Zhang, H.; Zheng, Y.; Li, M.; Ma, J.; Li, Y.; Chi, G. Lactate attenuates astrocytic inflammation by inhibiting ubiquitination and degradation of NDRG2 under oxygen-glucose deprivation conditions. *J. Neuroinflammation,* **2022**, *19*(1), 314. http://dx.doi.org/10.1186/s12974-022-02678-6 PMID: 36572898

[39] Xia, Q.; Zhan, G.; Mao, M.; Zhao, Y.; Li, X. TRIM45 causes neuronal damage by aggravating microglia-mediated neuroinflammation upon cerebral ischemia and reperfusion injury. *Exp. Mol. Med.,* **2022**, *54*(2), 180-193. http://dx.doi.org/10.1038/s12276-022-00734-y PMID: 35217833

[40] An, H.; Zhou, B.; Hayakawa, K.; Durán Laforet, V.; Park, J.H.; Nakamura, Y.; Mandeville, E.T.; Liu, N.; Guo, S.; Yu, Z.; Shi, J.; Wu, D.; Li, W.; Lo, E.H.; Ji, X. ATF5-mediated mitochondrial unfolded protein response (UPR^{mt}) protects neurons against oxygen-glucose deprivation and cerebral ischemia. *Stroke,* **2024**, *55*(7), 1904-1913. http://dx.doi.org/10.1161/STROKEAHA.123.045550 PMID: 38913800

[41] Wang, S.; Zhou, L.; Ji, N.; Sun, C.; Sun, L.; Sun, J.; Du, Y.; Zhang, N.; Li, Y.; Liu, W.; Lu, W. Targeting ACYP1-mediated glycolysis reverses lenvatinib resistance and restricts hepatocellular carcinoma progression. *Drug Resist. Updat.,* **2023**, *69*, 100976. http://dx.doi.org/10.1016/j.drup.2023.100976 PMID: 37210811

[42] Wang, S.; Wang, Y.; Yang, G.; Zhang, H.; Yuan, H.; Hou, C.; Zhao, L.; Suo, Y.; Sun, J.; Sun, L.; Lv, P.; Sun, Y.; Zhang, N.; Zhang, X.; Lu, W. Heat shock protein family A member 8 serving as a co-activator of transcriptional factor ETV4 up-regulates PHLDA2 to promote the growth of liver cancer. *Acta. Pharmacol. Sin.,* **2023**, *44*(12), 2525-2536. http://dx.doi.org/10.1038/s41401-023-01133-3 PMID: 37474643

[43] Kumar, P.; Nagarajan, A.; Uchil, P.D. Analysis of cell viability by the lactate dehydrogenase assay. *Cold Spring Harb. Protoc.,* **2018**, *2018*(6), pdb.prot095497. http://dx.doi.org/10.1101/pdb.prot095497 PMID: 29858337

[44] Decker, T.; Lohmann-Matthes, M.L. A quick and simple method for the quantitation of lactate dehydrogenase release in measurements of cellular cytotoxicity and tumor necrosis factor (TNF) activity. *J. Immunol. Methods,* **1988**, *115*(1), 61-69. http://dx.doi.org/10.1016/0022-1759(88)90310-9 PMID: 3192948

[45] Jurišić, V.; Spužić, I.; Konjević, G. A comparison of the NK cell cytotoxicity with effects of TNF-α against K-562 cells, determined by LDH release assay. *Cancer Lett.,* **1999**, *138*(1-2), 67-72. http://dx.doi.org/10.1016/S0304-3835(99)00011-7 PMID: 10378775

[46] Xie, X.; Wang, F.; Ge, W.; Meng, X.; Fan, L.; Zhang, W.; Wang, Z.; Ding, M.; Gu, S.; Xing, X.; Sun, X. Scutellarin attenuates oxidative stress and neuroinflammation in cerebral ischemia/reperfusion injury through PI3K/Akt-mediated Nrf2 signaling pathways. *Eur. J. Pharmacol.,* **2023**, *957*, 175979. http://dx.doi.org/10.1016/j.ejphar.2023.175979 PMID: 37611841

[47] Jian, W.; Ma, H.; Hu, Y.; Zhang, Q.; Xu, J.; Jiang, J.; Zhu, G.; Gong, Y. Oltipraz attenuated cerebral ischemia-reperfusion injury through inhibiting the oxidative stress and ferroptosis in mice. *Int. Immunopharmacol.,* **2024**, *140*, 112800. http://dx.doi.org/10.1016/j.intimp.2024.112800 PMID: 39096875

[48] Zhou, J.; Lei, Y.; Zhang, S.; Liu, Y.; Yi, D. Panaxadiol attenuates neuronal oxidative stress and apoptosis in cerebral ischemia/

reperfusion injury *via* regulation of the JAK3/STAT3/HIF-1α signaling pathway. *CNS Neurosci. Ther.,* **2025**, *31*(2), e70233. http://dx.doi.org/10.1111/cns.70233 PMID: 39957706

[49] Huang, Q.; Li, J.; Chen, J.; Zhang, Z.; Xu, P.; Qi, H.; Chen, Z.; Liu, J.; Lu, J.; Shi, M.; Zhang, Y.; Ma, Y.; Zhao, D.; Li, X. Ginsenoside compound K protects against cerebral ischemia/reperfusion injury *via* Mul1/Mfn2-mediated mitochondrial dynamics and bioenergy. *J. Ginseng Res.,* **2023**, *47*(3), 408-419. http://dx.doi.org/10.1016/j.jgr.2022.10.004 PMID: 37252276

[50] Yang, L.; Tao, Y.; Luo, L.; Zhang, Y.; Wang, X.; Meng, X. Dengzhan Xixin injection derived from a traditional Chinese herb Erigeron breviscapus ameliorates cerebral ischemia/reperfusion injury in rats *via* modulation of mitophagy and mitochondrial apoptosis. *J. Ethnopharmacol.,* **2022**, *288*, 114988. http://dx.doi.org/10.1016/j.jep.2022.114988 PMID: 35032588

[51] Juan, G.; Cavazzoni, M.; Sáez, G.T.; O'Connor, J.E. A fast kinetic method for assessing mitochondrial membrane potential in isolated hepatocytes with rhodamine 123 and flow cytometry. *Cytometry,* **1994**, *15*(4), 335-342. http://dx.doi.org/10.1002/cyto.990150409 PMID: 8026223

[52] He, Y.; Zheng, Z.; Liu, C.; Li, W.; Zhao, L.; Nie, G.; Li, H. Inhibiting DNA methylation alleviates cisplatin-induced hearing loss by decreasing oxidative stress-induced mitochondria-dependent apoptosis *via* the LRP1–PI3K/AKT pathway. *Acta. Pharm. Sin B,* **2022**, *12*(3), 1305-1321. http://dx.doi.org/10.1016/j.apsb.2021.11.002 PMID: 35530135

[53] Zhou, Z.; Li, W.; Ni, L.; Wang, T.; Huang, Y.; Yu, Y.; Hu, M.; Liu, Y.; Wang, J.; Huang, X.; Wang, Y. Icariin improves oxidative stress injury during ischemic stroke *via* inhibiting mPTP opening. *Mol. Med.,* **2024**, *30*(1), 77. http://dx.doi.org/10.1186/s10020-024-00847-2 PMID: 38840035

[54] Mendoza, A.; Patel, P.; Robichaux, D.; Ramirez, D.; Karch, J. Inhibition of the mPTP and lipid peroxidation is additively protective against I/R injury. *Circ. Res.,* **2024**, *134*(10), 1292-1305. http://dx.doi.org/10.1161/CIRCRESAHA.123.323882 PMID: 38618716

[55] Lissner, L.J.; Wartchow, K.M.; Toniazzo, A.P.; Gonçalves, C.A.; Rodrigues, L. Object recognition and Morris water maze to detect cognitive impairment from mild hippocampal damage in rats: A reflection based on the literature and experience. *Pharmacol. Biochem. Behav.,* **2021**, *210*, 173273. http://dx.doi.org/10.1016/j.pbb.2021.173273 PMID: 34536480

[56] Zhang, Y.; Li, T.; Pan, M.; Wang, W.; Huang, W.; Yuan, Y.; Xie, Z.; Chen, Y.; Peng, J.; Li, X.; Meng, Y. SIRT1 prevents cigarette smoking-induced lung fibroblasts activation by regulating mitochondrial oxidative stress and lipid metabolism. *J. Transl. Med.,* **2022**, *20*(1), 222. http://dx.doi.org/10.1186/s12967-022-03408-5 PMID: 35568871

[57] Iadecola, C.; Anrather, J. The immunology of stroke: From mechanisms to translation. *Nat. Med.,* **2011**, *17*(7), 796-808. http://dx.doi.org/10.1038/nm.2399 PMID: 21738161

[58] Duncombe, J.; Kitamura, A.; Hase, Y.; Ihara, M.; Kalaria, R.N.; Horsburgh, K. Chronic cerebral hypoperfusion: a key mechanism leading to vascular cognitive impairment and dementia. Closing the translational gap between rodent models and human vascular cognitive impairment and dementia. *Clin. Sci. (Lond),* **2017**, *131*(19), 2451-2468. http://dx.doi.org/10.1042/CS20160727 PMID: 28963120

[59] Zeng, J.; Xiao, H.; Li, X.; Liu, R-Z.; Zeng, Q.; Huang, Z-H.; Zhang, J-D.; Liu, Z-L. 3′-Daidzein sulfonate sodium protects against memory impairment and hippocampal damage caused by chronic cerebral hypoperfusion. *Neural. Regen. Res.,* **2018**, *13*(9), 1561-1567. http://dx.doi.org/10.4103/1673-5374.237119 PMID: 30127116

[60] Wang, D.P.; Lv, Q.L.; Lin, Q.; Kang, K.; Jin, K.Y.; Hai, J. The Cannabinoid receptor agonist WIN55,212-2 ameliorates hippocampal neuronal damage after chronic cerebral hypoperfusion possibly through inhibiting oxidative stress and ASK1-p38 signaling. *Neurotox. Res.,* **2020**, *37*(4), 847-856. http://dx.doi.org/10.1007/s12640-019-00141-8 PMID: 31808139

[61] Zhang, J.; Zhang, Y.; Zhang, Y.; Lu, K.; Zhang, X.; Wang, X.; Fu, J. Farrerol alleviates cognitive impairments in chronic cerebral hypoperfusion *via* suppressing NLRP3 inflammasome-mediated pyroptosis. *Int. Immunopharmacol.,* **2025**, *153*, 114442. http://dx.doi.org/10.1016/j.intimp.2025.114442 PMID: 40112599

[62] Xu, L.L.; Yang, S.; Zhou, L.Q.; Chu, Y.H.; Pang, X.W.; You, Y.F.; Zhang, H.; Zhang, L.Y.; Zhu, L.F.; Chen, L.; Shang, K.; Xiao, J.; Wang, W.; Tian, D.S.; Qin, C. Bruton's tyrosine kinase inhibition ameliorated neuroinflammation during chronic white matter ischemia. *J. Neuroinflammation,* **2024**, *21*(1), 195. http://dx.doi.org/10.1186/s12974-024-03187-4 PMID: 39097747

[63] Haupt, M.; Gerner, S.T.; Bähr, M.; Doeppner, T.R. Neuroprotective strategies for ischemic stroke—future perspectives. *Int. J. Mol. Sci.,* **2023**, *24*(5), 4334. http://dx.doi.org/10.3390/ijms24054334 PMID: 36901765

[64] Feske, S.K. Ischemic stroke. *Am. J. Med.,* **2021**, *134*(12), 1457-1464. http://dx.doi.org/10.1016/j.amjmed.2021.07.027 PMID: 34454905

[65] Li, C.; Sun, T.; Jiang, C. Recent advances in nanomedicines for the treatment of ischemic stroke. *Acta. Pharm. Sin B,* **2021**, *11*(7), 1767-1788. http://dx.doi.org/10.1016/j.apsb.2020.11.019 PMID: 34386320

[66] Zhu, T.; Wang, L.; Wang, L.; Wan, Q. Therapeutic targets of neuroprotection and neurorestoration in ischemic stroke: Applications for natural compounds from medicinal herbs. *Biomed. Pharmacother.,* **2022**, *148*, 112719. http://dx.doi.org/10.1016/j.biopha.2022.112719 PMID: 35168073

[67] Xu, W.; Yan, J.; Ocak, U.; Lenahan, C.; Shao, A.; Tang, J.; Zhang, J.; Zhang, J.H. Melanocortin 1 receptor attenuates early brain injury following subarachnoid hemorrhage by controlling mitochondrial metabolism *via* AMPK/SIRT1/PGC-1α pathway in rats. *Theranostics,* **2021**, *11*(2), 522-539. http://dx.doi.org/10.7150/thno.49426 PMID: 33391490

[68] Huang, J.; Liu, W.; Doycheva, D.M.; Gamdzyk, M.; Lu, W.; Tang, J.; Zhang, J.H. Ghrelin attenuates oxidative stress and neuronal apoptosis *via* GHSR-1α/AMPK/Sirt1/PGC-1α/UCP2 pathway in a rat model of neonatal HIE. *Free Radic Biol. Med.,* **2019**, *141*, 322-337. http://dx.doi.org/10.1016/j.freeradbiomed.2019.07.001 PMID: 31279091

[69] Velmurugan, G.V.; Vekaria, H.J.; Hartz, A.M.S.; Bauer, B.; Hubbard, W.B. Oxidative stress alters mitochondrial homeostasis in isolated brain capillaries. *Fluids Barriers CNS,* **2024**, *21*(1), 81. http://dx.doi.org/10.1186/s12987-024-00579-9 PMID: 39407313

[70] Yuan, Y.; He, Q.; Yang, X.; Flores, J.J.; Huang, L.; Luo, X.; Zhang, X.; Zhang, Z.; Li, R.; Gu, L.; Dong, S.; Zhu, S.; Yi, K.; Han, M.; Wu, L.; Zhou, Y.; Zhang, J.H.; Xie, Z.; Tang, J. Mitochondrial ferritin upregulation reduced oxidative stress and blood-brain-barrier disruption by maintaining cellular iron homeostasis in a neonatal rat model of germinal matrix hemorrhage. *Exp. Neurol.,* **2024**, *374*, 114703. http://dx.doi.org/10.1016/j.expneurol.2024.114703 PMID: 38281588

[71] Yang, J.L.; Mukda, S.; Chen, S.D. Diverse roles of mitochondria in ischemic stroke. *Redox Biol.,* **2018**, *16*, 263-275. http://dx.doi.org/10.1016/j.redox.2018.03.002 PMID: 29549824

[72] Kaur, M.M.; Sharma, D.S. Mitochondrial repair as potential pharmacological target in cerebral ischemia. *Mitochondrion,* **2022**, *63*, 23-31. http://dx.doi.org/10.1016/j.mito.2022.01.001 PMID: 34999014

[73] Wang, S.J.; Zhao, X.H.; Chen, W.; Bo, N.; Wang, X.J.; Chi, Z.F.; Wu, W. Sirtuin 1 activation enhances the PGC-1α/mitochondrial antioxidant system pathway in status epilepticus. *Mol. Med. Rep.,* **2015**, *11*(1), 521-526. http://dx.doi.org/10.3892/mmr.2014.2724 PMID: 25333348

[74] Chen, M.; Wang, Z.; Zhou, W.; Lu, C.; Ji, T.; Yang, W.; Jin, Z.; Tian, Y.; Lei, W.; Wu, S.; Fu, Q.; Wu, Z.; Wu, X.; Han, M.; Fang, M.; Yang, Y. SIRT1/PGC-1α signaling activation by mangiferin attenuates cerebral hypoxia/reoxygenation injury in neuroblastoma cells. *Eur. J. Pharmacol.,* **2021**, *907*, 174236. http://dx.doi.org/10.1016/j.ejphar.2021.174236 PMID: 34116043

[75] Liang, H.; Chang, X.; Xia, R.; Wu, W.; Guo, H.; Yang, M. Magnoflorine Attenuates cerebral ischemia-induced neuronal injury *via* Autophagy/Sirt1/AMPK signaling pathway. *Evid. Based Complement. Alternat. Med.,* **2022**, *2022*, 1-11. http://dx.doi.org/10.1155/2022/2131561 PMID: 36124014

[76] Li, H.; Liu, Q.; Zhu, C.; Sun, X.; Sun, C.; Yu, C.; Li, P.; Deng, X.; Wang, J. β-Nicotinamide mononucleotide activates NAD+/SIRT1

pathway and attenuates inflammatory and oxidative responses in the hippocampus regions of septic mice. *Redox Biol.,* **2023**, *63*, 102745.
http://dx.doi.org/10.1016/j.redox.2023.102745 PMID: 37201414

[77] Wu, H.; Wang, L.; Kang, P.; Zhou, X.; Li, W.; Xia, Z. The SP1/SIRT1/ACLY signaling axis mediates fatty acid oxidation in renal ischemia-reperfusion-induced renal fibrosis. *Int. Immunopharmacol.,* **2024**, *132*, 112002.
http://dx.doi.org/10.1016/j.intimp.2024.112002 PMID: 38608473

[78] Zhao, W.; Feng, S.; Wang, J.; Zhang, Z.; Chen, L.; Jiang, L.; Li, M.; Wang, T. Benserazide, a cystathionine beta-synthase (CBS) inhibitor, potentially enhances the anticancer effects of paclitaxel *via* inhibiting the S-sulfhydration of SIRT1 and the HIF1-α/VEGF pathway. *Front. Pharmacol.,* **2024**, *15*, 1404532.
http://dx.doi.org/10.3389/fphar.2024.1404532 PMID: 38828455

[79] Han, S.H.; Wu, M.; Nam, B.Y.; Park, J.T.; Yoo, T.H.; Kang, S.W.; Park, J.; Chinga, F.; Li, S.Y.; Susztak, K. PGC-1*α* protects from notch-induced kidney fibrosis development. *J. Am. Soc. Nephrol.,* **2017**, *28*(11), 3312-3322.
http://dx.doi.org/10.1681/ASN.2017020130 PMID: 28751525

[80] Dang, R.; Wang, M.; Li, X.; Wang, H.; Liu, L.; Wu, Q.; Zhao, J.; Ji, P.; Zhong, L.; Licinio, J.; Xie, P. Edaravone ameliorates depressive and anxiety-like behaviors *via* Sirt1/Nrf2/HO-1/Gpx4 pathway. *J. Neuroinflammation,* **2022**, *19*(1), 41.
http://dx.doi.org/10.1186/s12974-022-02400-6 PMID: 35130906

[81] Xu, J.; Wang, Y.; Wang, A.; Gao, Z.; Gao, X.; Chen, H.; Zhou, J.; Zhao, X.; Wang, Y. Safety and efficacy of Edaravone Dexborneol *versus* edaravone for patients with acute ischaemic stroke: A phase II, multicentre, randomised, double-blind, multiple-dose, active-controlled clinical trial. *Stroke Vasc. Neurol.,* **2019**, *4*(3), 109-114.
http://dx.doi.org/10.1136/svn-2018-000221 PMID: 31709115

[82] Rexrode, K.M.; Madsen, T.E.; Yu, A.Y.X.; Carcel, C.; Lichtman, J.H.; Miller, E.C. The impact of sex and gender on stroke. *Circ. Res.,* **2022**, *130*(4), 512-528.
http://dx.doi.org/10.1161/CIRCRESAHA.121.319915 PMID: 35175851

[83] Bushnell, C.D.; Chaturvedi, S.; Gage, K.R.; Herson, P.S.; Hurn, P.D.; Jiménez, M.C.; Kittner, S.J.; Madsen, T.E.; McCullough, L.D.; McDermott, M.; Reeves, M.J.; Rundek, T. Sex differences in stroke: Challenges and opportunities. *J. Cereb. Blood Flow Metab.,* **2018**, *38*(12), 2179-2191.
http://dx.doi.org/10.1177/0271678X18793324 PMID: 30114967
